# Predictive analytics of environmental adaptability in multi-omic network models

**DOI:** 10.1038/srep15147

**Published:** 2015-10-20

**Authors:** Claudio Angione, Pietro Lió

**Affiliations:** 1 Computer Laboratory - University of Cambridge, UK

## Abstract

Bacterial phenotypic traits and lifestyles in response to diverse environmental conditions depend on changes in the internal molecular environment. However, predicting bacterial adaptability is still difficult outside of laboratory controlled conditions. Many molecular levels can contribute to the adaptation to a changing environment: pathway structure, codon usage, metabolism. To measure adaptability to changing environmental conditions and over time, we develop a multi-omic model of Escherichia coli that accounts for metabolism, gene expression and codon usage at both transcription and translation levels. After the integration of multiple omics into the model, we propose a multiobjective optimization algorithm to find the allowable and optimal metabolic phenotypes through concurrent maximization or minimization of multiple metabolic markers. In the condition space, we propose Pareto hypervolume and spectral analysis as estimators of short term multi-omic (transcriptomic and metabolic) evolution, thus enabling comparative analysis of metabolic conditions. We therefore compare, evaluate and cluster different experimental conditions, models and bacterial strains according to their metabolic response in a multidimensional objective space, rather than in the original space of microarray data. We finally validate our methods on a phenomics dataset of growth conditions. Our framework, named METRADE, is freely available as a MATLAB toolbox.

As biologists would agree, there is no biology except in the light of evolution^1^. However, much of the uncertainty about the behavior of a microorganism is due to the lack of statistical bioinformatics methodologies for accurate measurement of adaptability to different environmental conditions and over time^2^,^3^. Approaches involving both mathematics and bioinformatics would benefit from the study of the molecular response to the adaptation. In turn, this would enable to discover the relation between the environmental (“external”) conditions and the changes in the metabolic-phenotypic networks (the “internal” environment). At the same time, it would elucidate the genotype-phenotype relationship, which is still an open problem in biology.

Many molecular levels can contribute to adaptability: (i) metabolism, i.e. the set of chemical reactions taking place in a living organism; (ii) pathway structure, namely groups of biologically-related reactions with a common goal; (iii) transcriptomics and codon usage, and in general the ability to regulate the speed of transcription and translation of genes into proteins. For instance, a highly adaptive bacterium ensures that the structure of its metabolism and the pathway productivity rapidly evolve over time due to varying environmental conditions or selective pressure^4^. Analogously, several recent examples show the coupling of codon usage to adaptive phenotypic variation, suggesting that the genotype functionality and behavior can be derived from the analysis of the evolution in the codon usage^5^. Typically, the correlation between gene expression and codon bias is large for environments similar to those in which the organism evolved, and small for dissimilar environments^6^.

Measurements of gene expression level are able to generate transcriptional profiles of microorganisms across a diverse set of environmental conditions. Databases of environmental conditions have been recently produced for several organisms, including *Escherichia coli*^7^, *Clostridium*^8^, *Salmonella*^9^, and fission yeast^10^. Although such resources, coupled with statistical analysis, remain key to the interpretation of measured data, they do not provide a comprehensive understanding of the resulting cellular behavior. Examples are the cases in which similar gene expression profiles may cause different phenotypic outcomes, while different environmental conditions may give rise to similar behaviors. Additionally, the actual response to a given condition is highly dependent on the multiple cellular objectives that the microorganism is required to meet^11^,^12^.

Here, we explore the adaptability of *E. coli* by investigating experimental conditions mapped to a multidimensional objective space. To obtain a phase-space of conditions, we add the gene expression and the codon usage layers to a flux-balance analysis (FBA) framework, therefore proposing a new multi-omic model. As a first result, we are able to optimize these layers for the overproduction of metabolites of interest, predicting the short term bacterial evolution towards the optimum. Then, we present a new method to map compendia of gene expression profiles to any metabolic objective space. Since each profile is associated with a growth condition, the objective space becomes the condition phase-space, which we investigate through principal component analysis, pseudospectra, and a spectral method for community detection.

To optimize these multi-omic layers, we propose a genetic multiobjective optimization algorithm that seeks the gene expression profiles such that multiple cellular functions are optimized concurrently. We use the Pareto front as a tool to seek trade-offs between two or more tasks performed by *E. coli*, and specifically to score the performance when the tasks are contending with one another. We simultaneously optimize tasks by finding the best gene expression profile and codon usage array. Most notably, this may permit to determine the best environmental condition in which a bacterium has to be grown in order to reach specific optimal output values from a range of objective functions chosen by the researcher. As a particular case, it is also possible to investigate the best single or multiple gene knockouts for the given set of objectives^13^.

The paper is organized as follows. First, we define the new multi-omic model by adding layers to FBA, in order to build the level of information required for a meaningful understanding of the landscape of experimental conditions. Using this augmented FBA framework, we optimize the model and we perform a temporal analysis of bacterial evolution towards an optimal configuration using the hypervolume indicator. Then, we introduce principal component analysis, pseudospectra and community detection methods to identify conditions mapped to close regions in the phase-space. We finally derive clusters of isoadaptability computed not in an absolute fashion, but taking into account the cellular multi-omic. Our approach is validated against a compendium of growth conditions including measurements of growth rates. The main steps of our pipeline, named METRADE (MEtabolic and TRanscriptomics ADaptation Estimator), are illustrated in Fig. 1.

The advantage of our approach is that it allows studying bacterial adaptability across multiple objectives (including biomass yield) in changing environmental settings, with the possibility to add available ‘omic data between gene expression and reaction rates by adjusting a continuous map. It requires only accurate information on the biochemical reaction system—provided by the full reaction list of the organism—and does not rely on knowledge of the kinetics of the system, which is usually missing. The key advantage of a continuous genotype-metabotype map is that one can reverse it, obtaining an enviromics map, which allows identifying the environmental factors leading to a pre-specified metabotype. To the best of our knowledge, very few approaches have been developed to take into account non-discretized gene expression levels in constraint-based models^14^, and none of them has integrated omic data with multi-objective Pareto analysis. Furthermore, for all we know, no prior studies have accounted for codons and combined Pareto-optimization with codon usage bias. The techniques included in METRADE (optimization, pseudospectra, component analysis and community detection of conditions) pave the way towards predicting and optimizing the bacterial adaptability across conditions and over time. METRADE is validated against a recently published phenomics compendium of growth conditions, and is made available in the Supplementary Information as a toolbox extension of COBRA 2.0^15^.

## Results

We derive a multi-omic model for the *Escherichia coli* able to account for the adaptability to multiple environmental conditions, and for the temporal evolution towards the production of selected metabolites. To build the multi-omic model, we map gene expression and codon usage to the metabolism by proposing a bilevel formulation that defines the flux bounds as a continuous function of the related expression data. We therefore generate a different model for each gene expression profile. This step is highly customizable in that it is possible to select a different function for each reaction in the model, thus allowing for the introduction of additional ‘omic data, e.g. protein localization or stochasticity in the protein abundance. The type of reaction-specific information that can generate a custom function for given enzymes is, for instance, the four-fold activity reduction in the isocitrate dehydrogenase enzyme when taking acetate as the carbon source^16^. In other words, we are able to generate a model tailored to any specific environmental or internal condition. The strain can be further optimized by finding the optimal codon usage for the given array of gene expression. The response to the environmental conditions is then mapped to an objective space and analyzed with statistical estimators. Finally, we propose a spectral method for community detection to infer sets of similar conditions according to the metabolic response measured in a multiobjective space.

### METRADE: a novel method to integrate and optimize gene expression and codon usage in FBA

Flux-balance analysis (FBA) is a mathematical approach based on linear algebra that enables the analysis of the flow of metabolites through a chemical reaction network^17^. According to FBA, a chemical reaction in the organism is associated with a flux in the model. Given *m* metabolites *X*_*i*_, *i* = 1, …, *m* and *n* reactions with flux rates *v*_*j*_, *j* = 1, …, *n*, the constraints are given not only by the lower and upper bound of the flux ranges (*capacity constraints*), but also by the balance of the concentration of the metabolites (*flux-balance constraints*). The balance that metabolites *X*_*i*_ must satisfy is


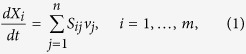


where *S*_*ij*_ is the stoichiometric coefficient of *X*_*i*_ in the *j*th reaction. Under steady state conditions 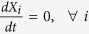, the balance for the i*th* metabolite is 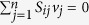. Therefore, at steady state, the balance equation is *Sv* = 0, where *S* is the stoichiometric matrix (*m* rows and *n* columns), and *v* is the vector of the fluxes (metabolic and transport fluxes).

Each reaction depends on a single gene set, represented by a string of genes linked by the AND/OR operators. For instance, when a gene set is composed by two genes in an “AND” relation, both are necessary to catalyze the corresponding reaction, and knocking out only one gene is sufficient to knock out the reaction. In this case, the gene set represents an *enzymatic complex*. Conversely, when a gene set is composed of two genes in an “OR” relation, the two genes synthesize for *isozymes*, which differ in the amino acid sequence, but catalyze the same reaction. Therefore, one gene is sufficient to catalyze the reaction, and both genes must be knocked out in order to knock out the reaction.

With the aim of overcoming the limitations offered by the Boolean knockout approach, we need to formalize the AND/OR relation between genes using a real function that makes it possible to define the *“gene sets expression”* as function of the gene expression. Let 
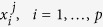
, be the gene expression levels of the genes 
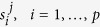
, and let 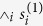 and 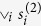 be two basic gene sets modeling an enzymatic complex and an isozyme respectively. We adopt the following map *τ* between a gene set and its expression:


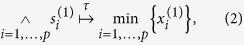



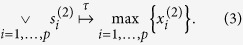


Specifically, the expression level of a gene set that needs all its genes to work properly, is constrained to be equal to the lowest of the expression levels of its genes. Conversely, the expression level of a gene set that needs at least one of its genes, is the highest of the expression levels of its genes. The bounds of a reaction catalyzed by an enzymatic complex will be function of the minimum expression level of its genes, while the bounds of a reaction catalyzed by an isozyme will be function of the maximum expression level of its genes. Nested gene sets are tackled using the same methodology, i.e. applying (2) and (3) recursively.

We run the model to find the distribution of fluxes that optimizes multiple metabolic markers (e.g., natural and synthetic objectives). As the bounds of the fluxes depend on the gene expression, we define the following bilevel linear program:


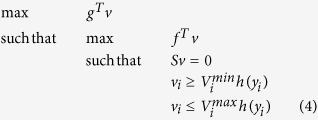


where *f* and *g* are *n*-dimensional arrays of weights associated with the first and second objectives respectively, and indicate how much the reaction fluxes *v* contribute to the objective function. 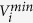 and 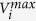 are the minimum and maximum flux of the wild-type configuration of the model. In the present study, *f* and *g* are Boolean vectors. For instance, *g*_*j*_ = 1 if and only if the flux *v*_*j*_ has to be maximized as second objective. In order to define the function *h*, let *y*_*i*_ be the gene set expression of the *i*th gene set, responsible for the *i*th reaction of the model. To map the gene set expression value into a specific condition of the model, we use the following piecewise multiplicative function:


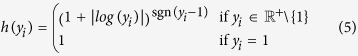


where 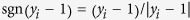.

In the FBA model, we replace the minimum and maximum flux of the *i*th reaction with 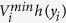 and 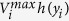 respectively. This choice is consistent with the fact that all the gene expression values in various conditions are relative to those of the wild-type bacterium. The gene expression is transformed by the logarithmic function *h*(⋅) so as to avoid that the genetic algorithm that we will use to perform the multiobjective optimization is driven towards high and unfeasible values of gene expression. This approach is in keeping with the “lazy step function” found in bacteria, yeast and human cells. According to this function, the mRNA levels are good indicators of the abundance of a protein (especially when averaging across populations), while post-transcriptional, post-translational and degradative regulations may fine-tune the protein abundance through miRNA^18^. Overall, in single living organisms, the correlation between mRNA level and protein abundance is good except when proteins are long lasting and mRNA short lived. In the latter case, the strength of the correlation is still a matter of debate. However, on large samples, the correlation has been shown to be evident with principal component analyses^19^ and especially for highly expressed genes, where the amount of noise is small and the correlation is high^6^,^20^,^21^. Furthermore, quantitative proteomics and transcriptome profiling (RNA-seq) seem to prove that there is high association between mRNA and protein levels, indicating that mRNA measures can be used as approximation for protein abundance^22^,^23^,^24^. More recently, Li *et al.*^25^ and Jovanovic *et al.*^26^ showed that mRNA levels are the main contributors to the overall protein expression level in mammals. In METRADE, we link the gene expression profiles with the FBA fluxes of the associated reactions in *E. coli* defining a real-valued adjustable map. Given that protein synthesis is an outcome of the expression of genes coding for protein segments, we link the gene expression values to the flux of the reactions controlled by the proteins coded by those genes.

While *h* is adjustable depending on the cell type or bacterial strain, we suggest a logarithmic map for a number of reasons. Specifically, the increase in the protein synthesis rate is fast with increasing mRNA abundance, but slower for large values of mRNA abundance^27^. Furthermore, adopting a logarithmic function in combination with the optimization algorithm avoids setting unrealistically high values of measured gene expression levels, which would be converted into weak constraints if using, e.g., a linear map. A key advantage of our map *h* is that it can be approximated by a linear function in the neighborhood of 1 (the wild-type gene expression level); this property models the experimental findings of high correlation and roughly linear relation between gene expression and enzyme activity for the wild-type *E. coli*^16^,^28^. Finally, several empirical evidences support our assumption^23^,^29^. We use the logarithmic map to set constraints for the metabolic model, then we solve the linear program (4) to find the flux distribution^30^.

Note that the outer maximization problem in (4) is subject to the inner one. More specifically, the inner maximization finds the distributions of flux in the network such that the growth rate is maximized. In the outer maximization, all the unregulated fluxes are then distributed such that the second objective 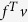 is maximized. The lower and upper bounds of the *i*th flux *v*_*i*_ depend on the expression level of the genes involved in the *i*th reaction. The bilevel problem is finally converted to a single-level problem^31^, and solved using the GLPK solver. It is straightforward to check that all the approaches based on Boolean gene knockouts become a particular case of METRADE, being 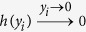 and *h*(1) = 1.

Then, we optimize the metabolic model through a multiobjective evolutionary algorithm, reaching an optimal configuration, according to the definitions in *Methods*. In the Boolean evolutionary approaches, each individual is a strain represented by a binary variable set representing the knockout strategy of gene sets^32^. Conversely, in METRADE the individuals are arrays of real values, each of which represents the expression level of a gene. We refer to these real-valued arrays as *gene expression arrays*. Through the function *h*(.), the gene expression arrays have a continuous effect on the FBA model, rather than only an on/off effect on reactions as in the Boolean approaches. Therefore, we are able to simulate cases where a lowly-expressed gene does not completely turn off the corresponding reaction (partial knockdown) and, analogously, a highly-expressed gene is able to increase the upper limit of the reaction flux (overexpression). For the multiobjective optimization, METRADE includes a parallel genetic algorithm (PGA) inspired to NSGA-II^33^ (see *Methods*).

**Figure 2:**
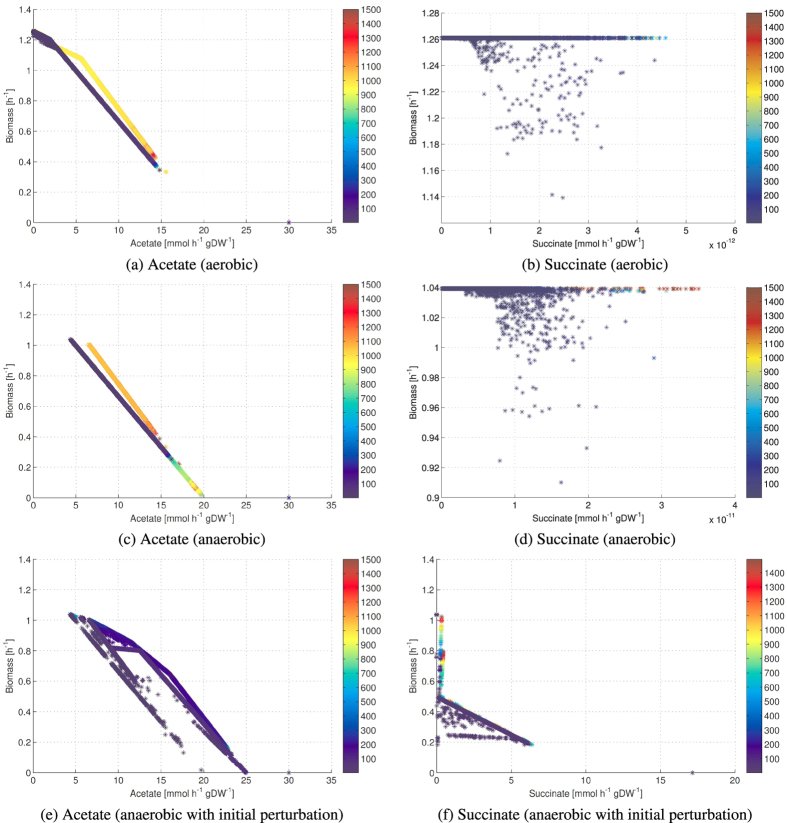
Pareto front produced by METRADE when maximizing succinate, acetate and biomass production through the parallel optimization algorithm. The optimization algorithm starts from an array of gene expression that, when translated into flux bounds, gives the default lower and upper bound of the initial model. On low succinate, slight variations of succinate flux cause step variations of biomass (plot (**f**)). The initial perturbation (**e**,**f**) is applied in anaerobic conditions on the first candidate strains and improves the convergence of the algorithm, thus permitting to avoid local maxima and to increase the coverage of the objective space. As a result, we discover a new area not explored by the algorithm applied in (**c**,**d**), including a new maximum for succinate production. This technique allows detecting the subspace where the bacterium operates (also called *metabolic potential*) in the objective space, and investigating scenarios of adaptability over time. Solutions are denoted by progressively warmer colors according to the time step of the PGA in which they have been generated adaptively from the starting point.

We test METRADE on the *i*JO1366 *E. coli* metabolic reconstruction^34^, consisting of three compartments (cytoplasm, periplasm and extracellular space), 1805 metabolites, 1366 genes, and 2583 reactions (including exchange and biomass reactions). The flux through the biomass reaction represents the rate at which the bacterium produces those metabolites necessary for its growth (e.g. amino acids, lipids, cofactors and proteins). The stoichiometry of the biomass reaction is scaled so that its flux rate equals the exponential growth rate of the bacterium. The objective functions taken into account are the fluxes representing the production of acetate, succinate, 1,2-propanediol and biomass (the major players in synthetic biology of *E. coli*). We start from a gene expression array equivalent to the case in which all the bounds of the fluxes are left unchanged with respect to the initial model (wild-type bacterium). In Fig. 2 we show the regions of objective space discovered by the genetic algorithm from the first to the last generation for anaerobic and aerobic conditions. As a case study, we maximize the acetate-biomass production, and succinate-biomass production. Both acetate and succinate are key molecules in biotechnology, with multiple industrial applications^35^.

In the anaerobic case, we also apply the same approach with a Gaussian noise added to the initial values of the gene expression arrays in order to avoid getting trapped in local maxima. Interestingly, in the acetate-biomass case (Fig. 2e), the area underlying the Pareto front is larger, and the number of optimal solutions is increased with respect to the case where no perturbations are applied. Furthermore, the Pareto front exhibits a curvature, although the extreme points (maximum acetate and maximum biomass) are conserved. In the succinate-biomass case, the same initial perturbation allows for a better coverage of the two-dimensional objective space (Fig. 2f), with a new extreme point of maximum succinate.

Without oxygen, the *E. coli* is able to grow with a maximum rate of 1.04 h^−1^, compared to 1.24 h^−1^ reached in presence of oxygen. Nevertheless, the production of succinate in anaerobic conditions is increased, especially when searching the optimal gene expression profile starting from the initial array with added noise. The gene expression profiles optimized towards maximum succinate production yields 17.14 mmol h^−1^ gDW^−1^ (millimoles per gram of dry weight per hour) but no biomass. A more interesting solution is 6.38 mmol h^−1^ gDW^−1^ of succinate with 0.18 h^−1^ of biomass. The maximum amount of biomass that can be achieved with a nonzero succinate production (0.34 mmol h^−1^ gDW^−1^) is 1.04 h^−1^.

A similar pattern emerges when maximizing acetate production and biomass. Specifically, in aerobic conditions, the maximum biomass is 1.26 h^−1^ and the maximum acetate is 15.56 mmol h^−1^ gDW^−1^ (not taking into account the extreme solution with no biomass). Conversely, in anaerobic conditions, the maximum biomass is 1.04 h^−1^ (with 4.36 mmol h^−1^ gDW^−1^ of acetate production), while the maximum acetate is 19.86 mmol h^−1^ gDW^−1^. Interestingly, both conditions share the same intermediate trade-off points with acetate production between 4.36 and 15.56 mmol h^−1^ gDW^−1^.

The main difference between anaerobic and aerobic conditions, especially when maximizing succinate as second objective, is the amount of succinate produced with an acceptable growth rate. The succinate in the cytoplasm takes part in 26 metabolic reactions. When no oxygen is imported, only five reactions are activated: succinate is a product of Succinyl-diaminopimelate desuccinylase, O-succinylhomoserine lyase, and Fumarate depended dihydroorotate, and a reactant of Succinate dehydrogenase and Succinyl-CoA synthetase. Ten transport fluxes are responsible for transferring succinate in the periplasm and in the extracellular space. In anaerobic conditions, the transfer is performed by proton antiport.

In the Supplementary Table we provide the points found by METRADE for the acetate-biomass and succinate-biomass maximization problems. We report the amount of natural (biomass) and second (acetate or succinate) objectives, the rank of the solution found by the PGA (0 if dominated, −1 if nondominated), the number of each individual and the generation in which the solution has been generated. Each row is associated with a specific gene expression profile found by the PGA.

### Optimization of codon usage in metabolic adaptation

The natural selection acts as a driving force at virtually all levels of the genetic information processing and biological organization: from the DNA stability, replication and transcription to messenger RNA life span and translation efficiency, to the correct functioning of the metabolic network in the building up and propagation of a living organism. Although, in principle, all these constraints could interact in a very complex way, it is indeed fruitful to try to untangle the role of each element.

The process of translation of the genome allows expressing genes into cellular functions. The translation of coding sequences into proteins starts when the ribosome is positioned on the AUG codon (except for some genes using alternative start codons), followed by the polypeptide synthesis in the ribosomal tunnel. The rate of protein synthesis depends on many factors, e.g. the rate of transfer RNA (tRNA) binding and the kinetics of the process. Each tRNA provides the code to assign a triplet of nucleotides (*codon*) to a specific amino acid. A tRNA exposes an amino acid and a nucleotide triplet (*anticodon*) that recognizes a specific codon. Specifically, there are 20 amino acids and 4^3^ codons, 61 of which actually encode for amino acids. Since the sense codons are more than the amino acids, an amino acid will be encoded by one or more codons, while each codon encodes always for the same amino acid^36^. In particular, an amino acid of a growing polypeptide chain can be encoded by up to six codons. Codons coding for the same amino acid are referred to as *synonymous codons*.

The different tRNA species exposing the same amino acid, and therefore associated with synonymous codons, are differentially expressed: some tRNAs are more abundant than their synonymous cognates. As a consequence, synonymous codons are not equivalent and are not used randomly. The codon bias is strongest in highly expressed genes, indicating that codon composition has an impact on translation efficiency^37^. Specifically, high-frequency-usage codons allow the quick generation of the polypeptide chain, while low-frequency-usage codons slow down the translation process and allow the nascent protein to fold into a helical structure^38^,^39^. In this regard, it has been experimentally proved that replacing rare codons with frequent synonymous codons improves the rate of translation^40^. The usage of each codon reflects the amount of its corresponding tRNA. Differences in codon usage frequency are therefore responsible for rapid or slow translation of genes into proteins, thus affecting the final gene expression^41^. The codon usage in bacteria can modulate the translation to reach the maximum rate of 15 amino acids per second on average.

While FBA and flux optimization capture the behavior of an organism at steady state, controlling and optimizing the codon usage may also allow to capture also the phenotypic noise^42^, therefore permitting the adaptation of the organism to a variety of environments. An optimal codon usage also enables fast translation without misincorporations. Recently, optimization and evolutionary steps have been coupled taking account of subsequent engulfments leading to specialization. In this regard, the Pareto front has been proposed as a tool to shed light on the putative evolution from the ancestor bacterium to the full functionality of an organelle, through a number of adaptive evolutionary steps^43^. In this study we assess the genome-wide transcriptional and translational organization by analyzing the multiobjective optimization of the codon usage distribution in genes, and how this affects the fluxes in the same pathway. By using perturbations of fluxes and codon bias, one can simulate the evolution of a non-synchronized population of bacteria as alternated phases of exponential growth (feast) and selection (famine). During the sporadic feast periods, the number of bacterial cells tends to increase exponentially and the competition between phenotypes develops very harshly, resulting in a large predominance of the fittest genotype. By modeling the codon bias in the FBA framework, we establish estimators (Pareto optimality and associated measures) of the transcriptional and the translational fitness bottlenecks in metabolic pathways. This represents a guide for practical solutions of synthetic biology for gene design in natural strains.

### Accounting for codon usage in the FBA framework

Manipulating and co-optimizing the gene expression and the codon usage of a bacterium may allow overproducing relevant compounds, from therapeutic and industrial standpoints (e.g., amino acids and alcohols)^44^. This is of key importance when aiming at producing desired products through biosustainable processes. The idea underlying our approach is that even if two gene expression profiles are identical, the organism has the possibility to optimize its codon usage with small variations, allowing a co-optimization for a given set of objectives.

To account for codon usage frequency in the translation process, we analyze a simplified situation where genes are made up of a slow codon *c*^(1)^ and a fast codon *c*^(2)^, read by two tRNAs with abundance *a*^(1)^ and *a*^(2)^ respectively^45^,^46^. Let us denote by 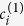 and 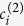 the slow and fast codon usage of the *i*th gene. In each gene *g*_*i*_, the codons 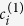 and 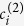 can be used independently from one another. Since the total usage of a codon *c*^(*j*)^ by all the genes *g*_*i*_ depends on the abundance *a*_*j*_ of the corresponding tRNA, additional constraints in our model are


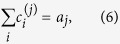


where *j* = 1,2 ranges over the codons, and *i* ranges over the genes.

In order to optimize the codon bias, we let the codon usage values 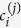 evolve using the PGA, and the constraints (6) hold at each generation of the algorithm. Then, the codon usage bias influences the rate of protein synthesis, and therefore the related reaction flux. If the fast codon is used more than the slow one, the translation process becomes faster. The production of the protein will be boosted by fast codons also because more ribosomes could operate on the same mRNA. To achieve this, the value *y*_*i*_ representing the gene set expression in METRADE is modified accordingly, thus obtaining a final variable *z*_*i*_ that includes the effects of the gene expression and the codon usage bias:


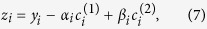


where 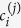 are the values representing the codon usage (variables for the multiobjective optimization), while *α*_*i*_ and *β*_*i*_ are multiplicative constants that can be used to increase the effect of the slow codon with respect to the fast one, or vice versa. We set 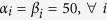, so as to obtain a noticeable effect of the codon usage even with a small number of generations from the optimization algorithm (with different values of *α*_*i*_ and *β*_*i*_ we obtained different speed of convergence but the same shape of Pareto front). These two parameters and the equation (7) can be used to adjust the strength of the codon bias on the overall protein synthesis. Finally, we use (5) applied to *z*_*i*_ to compute the flux bounds, and (4) to compute the flux distribution. In this formulation, we do not take into account clusters and positions of rare codons. The definition (7) models the effect that the codon usage bias has on the final expression, and mimics the fact that the codon usage strongly affects translation rate and the protein production^47^. In this regard, it has been recently shown that a change in the synonymous codon usage in an N-terminal peptide may result in an increase in the protein abundance up to 60-fold^48^.

**Figure 3:**
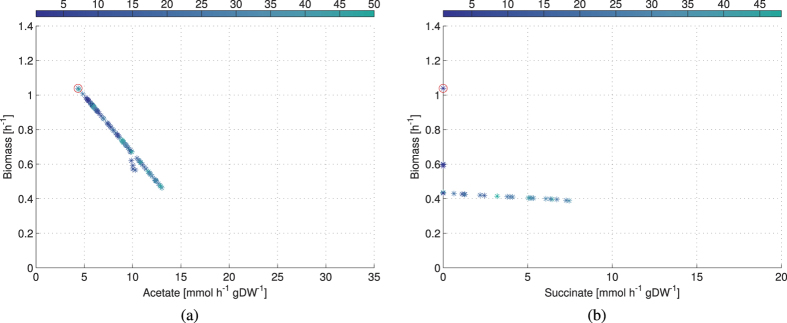
Optimization of codon usage for simultaneous maximization of acetate and biomass production (**a**), and succinate and biomass production (**b**) starting from a wild-type *E. coli*. The Pareto front is obtained by applying our optimization routine to the variables representing the codon usage. We let METRADE run for 50 generations, which a preliminary analysis proved sufficient to obtain a Pareto front spanning the objective space. We denote solutions by progressively lighter colors depending on the generation in which they have been found. The red circle corresponds to the fixed array of gene expression levels associated with the wild-type *E. coli* strain.

In Fig. 3, we present the optimization of acetate, succinate and biomass production starting from a wild-type *E. coli* strain and using the codon usage values as variables (individuals) of the PGA. In the acetate-biomass optimization, the acetate production increases linearly with decreasing biomass. In the succinate-biomass optimization, a slight change in the codon usage towards an increased succinate production causes the growth rate to drop from 1.04 h^−1^ to 0.60 h^−1^. A further drop (0.39 h^−1^) allows producing 7.45 h^−1^ gDW^−1^ of succinate.

### Mapping genotype-phenotype associations to multidimensional objective spaces

Another useful feature of METRADE is the possibility to map a gene expression microarray profile directly to a bidimensional space of objective functions (e.g., acetate, biomass and succinate). Compared to Boolean associations between gene presence/absence and reaction activation/inactivation, this feature is extremely useful in the sense that it allows continuous modulation of the output. As a result, even the effect of small variations of gene expression level is captured, and could have a significant impact on the metabolism. Here we use a compendium of 466 *E. coli* Affymetrix Antisense2 microarray expression profiles^7^. The dataset includes data collected in different media and different conditions, such as pH changes, antibiotics, heat shock, varying glucose and oxygen concentrations.

The idea underlying our approach is that each condition yields a particular gene expression profile, which we convert into constraints for the FBA model in order to evaluate the condition-specific metabolic response. After defining the first and second objectives, we run the model and, for each condition, we obtain a point in the selected objective space. The model is run with an oxygen and glucose intake rate depending on the oxygen and glucose of the condition in which the bacterium was grown.

We assume that the genetic level is slower than the metabolic one, and therefore the steady state is reached faster than the variation of enzyme concentrations due to changes in the gene expression profile^49^. As a result, the metabolism is assumed to be always at a steady state that depends on the environmental factors. In this way, the gene expression data are used as estimator of the activity of the corresponding reaction in the model. We are therefore able to associate a given gene expression profile with a single point in a multidimensional and user-defined objective space.

Furthermore, we take into account that a gene whose expression level is only slightly varied across conditions is a key gene for the organism^50^. The importance of a gene - and therefore the robustness of the reaction fluxes for which that gene is responsible - is inversely proportional to its variance across all the experimental conditions. Then, we solve the bilevel problem (4) replacing the function *h* with the function 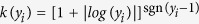, where 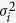 is the variance of the gene set responsible for the *i*th reaction, and *γ* is a weight for the variance. The variances 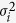 of the gene sets are computed from the variances of the genes across the conditions in the dataset, following the same rules defined to map the gene expressions to the gene set expressions (Equations (2) and (3)).

**Figure 4:**
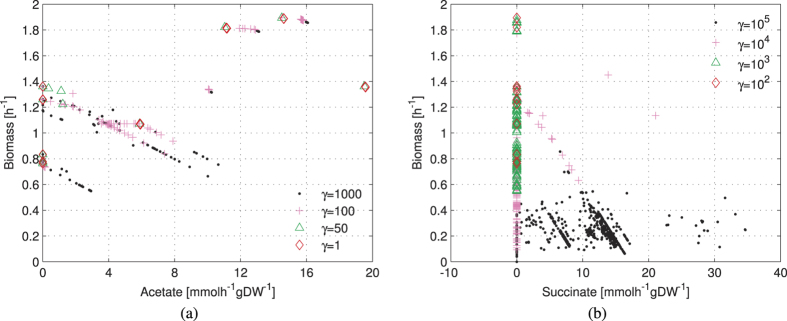
The 466 profiles of gene expression (each associated with one condition) positioned in the two-dimensional space acetate-biomass (**a**) and succinate-biomass (**b**). The parameter *γ* represents the weight attributed to the variance as an indicator of the importance of a gene, and determines the effect of the gene expression values on the final reaction rates. By increasing the parameter *γ* we increase this effect, therefore even two experimental conditions with slight differences in the gene expression profiles are mapped to different points in the objective space. The map from genes to metabolism is robust with respect to perturbations of *γ*, while large perturbations of *γ* (orders of magnitude) increase the sensitivity of the metabolism to the different environmental conditions.

As case-studies, we choose the acetate-biomass space (Fig. 4a) and the succinate-biomass space (Fig. 4b). For increasing *γ*, the *E. coli* is able to move towards the production of the second objective rather than the natural objective. For the succinate-biomass case, the best trade-off is reached when *γ* = 10^4^: the best gene expression profiles are able to produce 21.06 and 13.87 mmol h^−1^ gDW^−1^ of succinate with a biomass of 1.13 and 1.45 h^−1^ respectively. Nevertheless, an excessive role attributed to gene expression as a multiplicative factor for the flux bounds (e.g. *γ* = 10^5^ in the succinate-biomass space) leads to a reduced production of biomass (0.85 h^−1^ maximum), although providing remarkably high values of flux rates for the second objective (up to 34.67 mmol h^−1^ gDW^−1^). Conversely, for the acetate-biomass case, increasing *γ* improves the area of the space covered, but does not provide remarkable new regions of increased acetate and biomass yield. We also increased the order of magnitude of *γ* over 10^3^, but we did not notice significant changes with respect to *γ* = 10^3^.

While gene expression maps the external or internal condition of the organism, the codon usage maps quick alterations to fine tune the amount of protein produced. A possible application of the gene expression and codon usage co-optimization is to evaluate the ratio between the gene expression and the codon usage variations with respect to a given non-optimal condition. This ratio can be computed for every pathway and exploited to highlight the difference among pathways. As a result, the Pareto front becomes a promising tool to investigate a model from a multi-omic standpoint.

### The hypervolume indicator as a measure of adaptation over time

**Figure 5:**
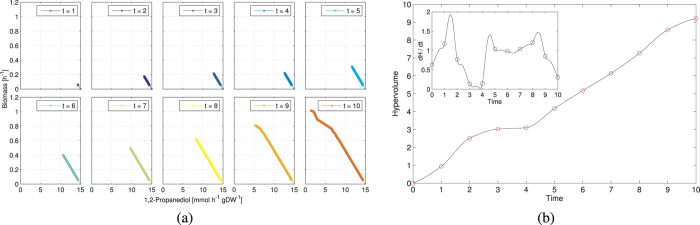
Temporal evolution of *E. coli* K-12 MG1655 grown on MOPS minimal medium when optimizing concurrently towards 1,2-propanediol production and growth rate using the PGA. (**a**) Evolving tradeoff towards the optimal production rates. (**b**) Hypervolume indicator over time. The hypervolume shows a plateau between two phases of increase. In the inset, the first derivative of the hypervolume correctly highlights the alternating periods of slow and fast evolution. The discrete time points have been interpolated with a cubic piecewise polynomial.

During the growth process of a bacterial population, the short term evolution of a single strain ranges from a wild-type configuration towards an optimized configuration where multiple objectives are taken into account. In order to gain insights into the evolution of an *E. coli* strain on a short temporal scale, and provide a more accurate description of its order of growth, we analyze the dynamics of the strain on a bidimensional objective space consisting of biomass and 1,2-propanediol production in anaerobic conditions (Fig. 5a). Different strains may evolve on large temporal scales on the same bidimensional space, starting from different initial points. Here we take into account the initial point that refers to the wild-type *E. coli* K-12 MG1655 grown on morpholinepropanesulfonic acid (MOPS) minimal medium, with anaerobic aeration, culture temperature of 37 °C and 11 mM of glucose. The full Pareto front and the dominated points are shown in Fig. S1.

In order to investigate the evolving tradeoff in the objective space, we use the Pareto front as a measure of the evolution of the strain. Specifically, we quantify the evolution by computing the hypervolume indicator of the process, and its first derivative. The hypervolume is an indicator for the size of the space covered by the Pareto front in a multidimensional objective space (see *Methods*). Among its properties, it is strictly monotonic with respect to strict Pareto dominance. It follows that the ideal Pareto front, reached asymptotically when the number of populations generated approaches infinity, achieves the maximum hypervolume available for the system^51^.

We propose the hypervolume as a proxy for the versatility of an organism and for its ability to ensure simultaneous production of multiple chemicals. A wild-type bacterium is specialized towards the production of biomass only, therefore lying on one axis of the multidimensional space, i.e. with null hypervolume. During the evolution towards multiple objectives, the bacterium moves and covers increasing portions of the objective space. The size of the space covered can be measured with the hypervolume indicator, while the speed of evolution can be associated with the hypervolume’s first derivative. In Fig. 5b, we plot the evolution of the hypervolume indicator over time for the K-12 MG1655 *E. coli* that moves on the objective space towards maximization of 1,2-propanediol production and growth rate. We divide the evolution time into 10 time steps. The initial growth ends after 2 time steps, and starts again after 4 time steps, decreasing at the final step. The derivative of the hypervolume indicator highlights alternating periods of slow and fast evolution that could be associated with the feast and famine growth phases of the bacterial population.

### Principal component analysis on multiobjective spaces reveals genotype-phenotype relationship

**Figure 6:**
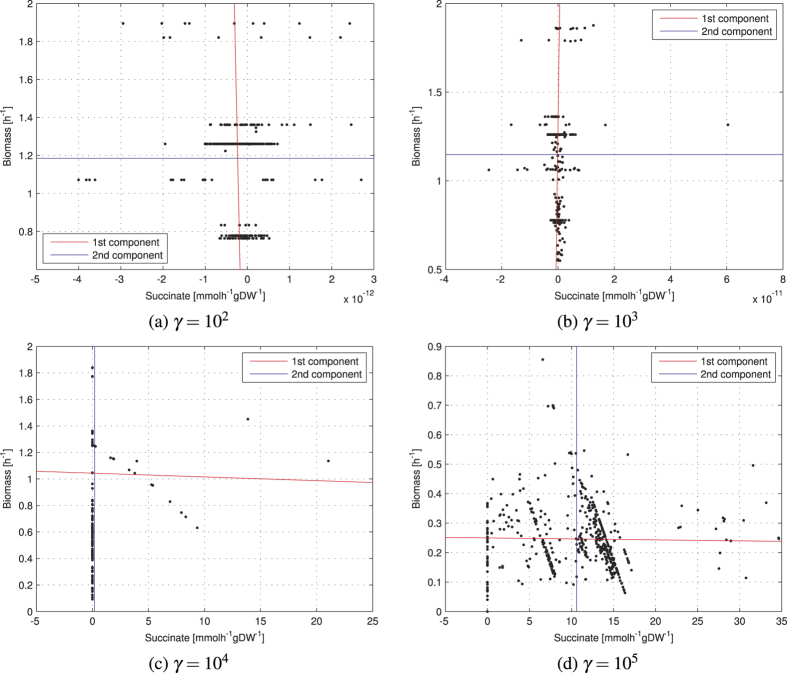
PCA in the succinate-biomass objective space. The two principal components are computed using the principal component analysis on the centered data (*x* − *μ*_*x*_, *y* − *μ*_*y*_), but are plotted on the original data. The slope of the first principal component (i.e., the direction of maximum variance of the data), depends on the parameter *γ*, the multiplicative factor that influences the effect of the gene expression data on the upper and lower bounds of the reaction fluxes in the multi-omic FBA model. The second principal component is always perpendicular to the first component (not highlighted in these plots due to different scales used). With respect to the first two cases (top), the direction of the first principal component in the last two cases (bottom) is remarkably different, as shown in Table 1.

In the objective spaces obtained when mapping the gene expression profiles to the target metabolites, we perform a principal component analysis (PCA) based on the singular value decomposition (SVD) of the data obtained by mapping each gene expression condition on the objective space. In our case, PCA is applied to a bidimensional objective space to analyze how solutions are distributed in the space (Fig. 6 for succinate-biomass, Fig. S2 for acetate-biomass). This is equivalent to finding the system of axes in which the covariance matrix is diagonal. PCA is often used to detect redundancy of information due to the fact that group of variables may vary together, and therefore can be replaced by a single variable. This is achieved through the definition of new variables as linear combinations of the original variables. The new variables, called *principal components*, are orthogonal to each other (so as to avoid redundancy) and represent an orthogonal basis. The simplification is achieved by discarding those components that explain little variance in the data, i.e. the components corresponding to the smallest eigenvalues of the covariance matrix.

We apply PCA to the points representing the *E. coli* conditions mapped to the bidimensional objective space. The eigenvalues *l*_1_ and *l*_2_ of the covariance matrix indicate the variance explained by the first and second principal components respectively. The eigenvector with the largest eigenvalue *l*_1_ represents the direction of maximal variation of the points in the objective space. Combining PCA and the map between gene expression profiles and multidimensional objective spaces allows assessing the relative impact of *γ* on the position of each gene expression profile in the objective space, and merits further experimental investigation (e.g., with a parameter optimization algorithm). More specifically, our results show that the parameter *γ*, which represents the effect of the gene expression on the final reaction bounds of the multi-omic model, has a direct effect on the direction of the maximum variance, as shown in Table 1. We note that, while in two dimensions PCA can be generally avoided and in some cases replaced by visual inspection, the combination between METRADE and PCA is useful when optimizing for more than two objectives. Using PCA, the dimensionality of the phase-space of conditions can be reduced by looking at the directions of “phenotypic” maximum variance across a given set of environmental conditions.

### Community structure in condition-dependent bacteria using spectral methods

The similarities between phenotypic outcomes of a set of environmental conditions are highly dependent on the objectives that the bacterium is required to maximize. To further study the relation between the 466 microarray profiles when mapped to a multiobjective space, we build a binary network whose adjacency matrix *D* depends on the Euclidean distances between the points in the acetate-biomass and succinate-biomass objective space.

First, in each bidimensional objective space, we compute a 466 × 466 real-valued matrix of distance


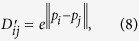


where *p* is an ordered pair indicating the coordinates of the associated point in the objective space. Then, we obtain the binary network by fixing a threshold *η* and adopting a “nearest neighbors” approach: the link between node *i* and node *j* in 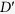 is kept in *D* if and only if 
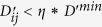
. We set 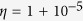 to ensure that only points (i.e., conditions) close to each other are part of the same connected component. As a result, the network is sparse. To detect communities in this sparse network, we first divide it into connected components, indicating points close to each other in the objective space. Then, we use the flow matrix (12) and a spectral method for community detection to further investigate each component (see *Methods*). The community structure predicted by our framework in the biomass-acetate and in the biomass-succinate cases are reported as Supplementary Information. Interestingly, the algorithm classifies conditions in different ways according to the objectives chosen for the optimization of the metabolism. For instance, the conditions hscA_U_N0075 and gyrI_U_N0075 belong to the same community if the *E. coli* is optimized towards the production of succinate and biomass, therefore indicating similar response, but belong to different communities when the bacterium is optimized for acetate and biomass production. This is in keeping with the fact that the objectives to maximize also shape the bacterial metabolic response to the changing environment. (The reader is referred to the Supplementary Information also for details on the communities and on the experimental settings of each condition.)

**Figure 7:**
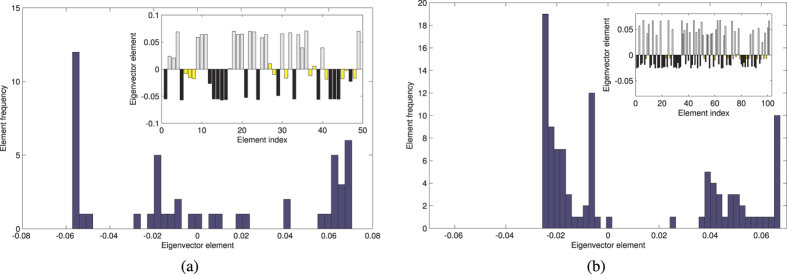
We build a flow matrix *F* of the largest connected component of *D*, and we plot the components of its Fiedler eigenvector. The sign of the components of the Fiedler eigenvector indicates the community to which nodes (i.e. conditions) belong^80^. In finding community structures, the algorithm performs better in the acetate-biomass case (**a**), where it shows a bimodal distribution, rather than in the succinate-biomass case (**b**). For the latter case, a possible correction could be to move the community assignment threshold towards values greater than 0.

Let us consider the largest connected component of *D*. We compute its flow matrix *F* to investigate communities. The histograms we obtain (Fig. 7) indicate that the spectral method (for community detection) performs generally well on our matrices, since is able to clearly assign the vast majority of nodes (conditions) to communities. This is in line with the results obtained by Newman^52^, and confirms that spectral methods applied to the flow matrix are able to classify nodes of sparse networks, where standard spectral methods are often unable to find communities.

We studied the degree distribution and the properties of this network through the spectral method. The algorithm is able to find communities in the acetate-biomass space better than in the succinate-biomass space, meaning that the same compendium of experimental conditions yields points close to each other in the latter objective space. This is confirmed by the fact that the largest connected component in the succinate-biomass space is composed of 101 nodes (only 49 in the acetate-biomass space). These results indicate that, in the same set of experimental conditions, it is more challenging to differentiate the behavior of the *E. coli* metabolism when it is optimized for succinate and biomass production.

**Figure 8:**
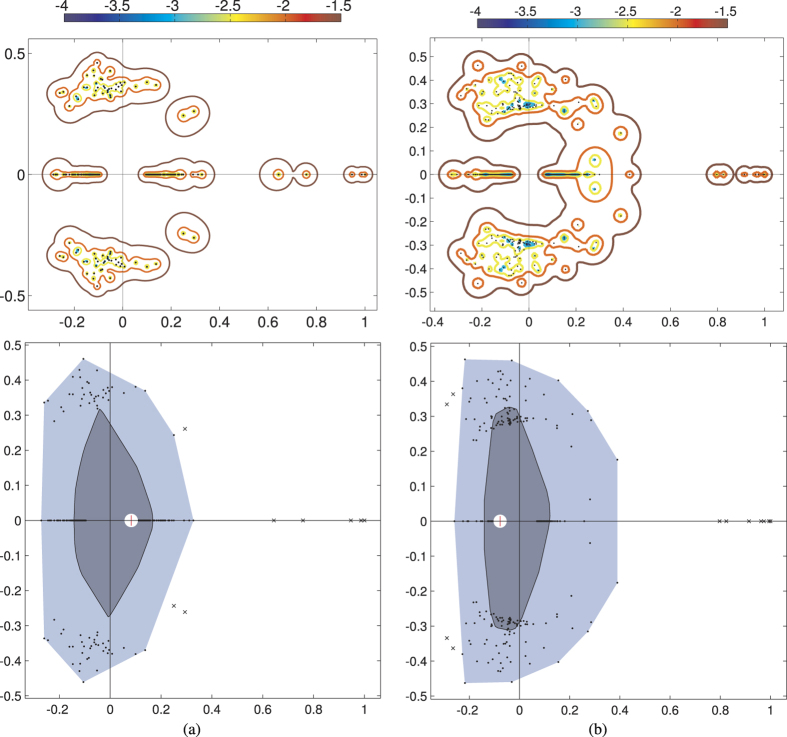
Pseudospectra of the flow matrix computed on the largest connected component of *D* with *ε* ranging from 10^−4^ to 10^−1.5^. (**a**) Acetate-biomass space: for *ε* = 10^−1.5^, around the three leading real eigenvalues, the pseudospectrum is a connected set, while for *ε* = 10^−2^ and smaller values it consists of disjoint sets. (**b**) Succinate-biomass space: the largest connected component is bigger, indicating that the experimental conditions yield points close to each other in this output space. There is a clear distinction between the seven leading real eigenvalues and the rest of the spectrum. The bagplot (bottom) shows that the median eigenvalue (red “plus” sign) is remarkably different in the two cases: in the succinate-biomass space, it has a negative real part. The eigenvalues are closer to each other in the succinate-biomass space, since the inner bag (dark grey) containing the 50% of them is less spread than in the acetate-biomass space.

In order to gain insight into the distribution of the position of each condition in the objective space in presence of uncertainty due to external perturbations on gene expression data, in Fig. 8, we plot the *ε*-pseudospectrum Λ_*ε*_ of the flow matrix *F*, consisting of the spectra Λ of a matrix approximating *F* with an error matrix *E* with negligible norm *ε*^53^. Formally, the pseudospectrum of *F* is defined as:


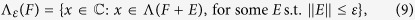


or equivalently, as 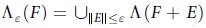. To perform a visual cross-comparison between different spectra in the complex plane of Fig. 8 we adopt the bagplot (the bivariate equivalent of the boxplot)^54^. The main components of the bagplot are: the *bag*, a convex set which contains 50% of the eigenvalues, and the *fence*, which separates inliers from outliers. While the fence is usually not included in the graphical representation of the bagplot, the points in the fence but not in the bag are part of a light grey *loop*, the convex hull of all the points inside the fence. The bagplot proves useful to explore the median and shape of the inner bag for cross-comparison purposes between the different sets of objectives optimized by the microorganism.

Given the shape of the connected components in the objective space, the detection of community structures is crucial when visual inspection of the objective space is not sufficient. In fact, this method is key to determining the specific objective (acetate, succinate or biomass) that is best obtained in every community of conditions, and can be used to shed light on the communities of conditions when given phenotypic properties are required. Therefore, on a Pareto front it can be readily used in the decision making process to select those point that are aimed at the maximization of a specific objective.

### Validation of METRADE on a compendium of phenomics data

**Figure 9:**
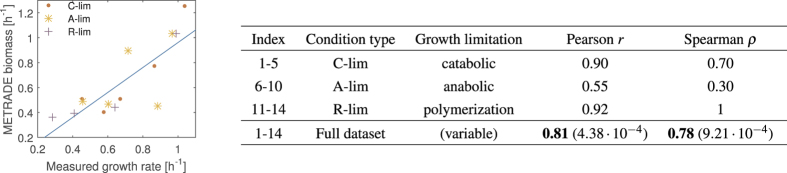
(Left) METRADE predictions and measured growth rates for each subset of the dataset used in this study. The subsets of conditions are denoted by (i) *C-lim*: titrated catabolic flux through controlled inducible expression of the lacY gene; (ii) *A-lim*: titrated anabolic flux through controlled expression of GOGAT; (iii) *R-lim*: inhibition of protein synthesis with an antibiotic (chloramphenicol). (Right) Spearman’s *ρ* and Pearson’s *r* correlation coefficients between METRADE and experiments. The *p*-value is reported in brackets. While obtaining a good overall correlation (final row of the table) between experimentally measured growth rates and the biomass predicted by METRADE, we obtain the best results in the conditions representing inhibited protein synthesis by supplying chloramphenicol to the growth medium. The full set of gene expression profiles, as well as our results and predictions are reported as Supplementary Information.

To validate METRADE, we use the phenomics dataset by Hui *et al.*^55^. The compendium contains 14 expression profiles in different growth conditions, with measured growth rates between 0.28 and 1.04 *h*^−1^. The different conditions were obtained with: (i) titrated catabolic flux through controlled inducible expression of the lacY gene; (ii) titrated anabolic flux through controlled expression of GOGAT; (iii) inhibition of protein synthesis with an antibiotic (chloramphenicol). Overall, we obtain remarkable results in predicting the growth rate from the expression profile associated with each condition. On the full dataset, we obtain a strong correlation between predicted and measured growth rates (Pearson’s *r* = 0.81, *p*-*value* = 4.38 · 10^−4^, and Spearman’s *ρ* = 0.78, *p*-*value* = 9.21 · 10^−4^). The best results are obtained with the subset of conditions representing inhibited protein synthesis by supplying chloramphenicol to the growth medium (Pearson’s *r* = 0.92, Spearman’s *ρ* = 1). A comparison between predicted and measured growth rates is reported in Fig. 9 and Fig. S3. The full dataset, the growth rates measured experimentally and those predicted by METRADE are reported as Supplementary Information.

Changing flux rates *in vitro* or performing gene overexpression and partial knockdown suggested by METRADE is less straightforward than performing gene knockout. However, the number of techniques to perform gene expression changes in bacteria is rapidly increasing. The methods available to date have been recently reviewed by Yen *et al.*^56^. Our predictions on overexpression or partial gene knockdown can be implemented using plasmids or through promoter engineering based on CRISPR-Cas, homologous recombination or transposable elements, RNA programming devices^57^, and engineering of ligands to create sensors for regulation of gene expression^58^. Modulation of gene (or protein) expression level can be achieved through engineering of ribosomal binding sites^59^,^60^,^61^ and promoters^62^,^63^,^64^.

## Discussion

Bacterial adaptability to new environmental conditions involves shifts in the gene expression levels and in the biochemical network, also in places that are not always related directly with the adaptation. For instance, an increase in the amount of a given nutrient supplied to the bacterium will increase the request for enzymes—and consequently for production of reactants—able to produce that nutrient^65^. Although increasing research efforts have been allocated in the attempt to understand the relation between gene expression changes and phenotype in bacteria, little is known about the contribution of the different omics and different objectives to the phenotypic adaptability and evolution.

Our study describes a framework named METRADE, and is structured as follows. First, we derive a novel multi-omic FBA model by implementing and combining multiple target optimization with gene expression and codon usage as additional layers of the most complete metabolic model available for *Escherichia coli*^34^. Each point of the Pareto front represents a different strain or the same strain adapting differently to various sets of environmental conditions. Given an *E. coli* with a specific gene expression configuration, METRADE is able to determine the best codon usage for each gene so as to maximize or minimize desired objective functions.

Then, we use METRADE to map environmental conditions to the variation of the gene expression, and we investigate the effect on the phenotype (through the metabolic map). Our method associates each gene expression profile with a flux distribution represented by a point in a multidimensional objective space, which therefore becomes a condition phase-space. Then, we focus on the condition phase-space and we propose a set of techniques to analyze the adaptability to experimental conditions. The principal component analysis allows exploring the directions with largest variance, indicating the relation between the gene expression profiles and the variance in the two or more objectives chosen. The hypervolume indicator used within METRADE is focused on how the trade-off evolves in a bidimensional objective space, highlighting periods of slow and fast evolution. Finally, the spectral method for community detection, the pseudospectrum and its bagplot are used to further analyze the experimental conditions and to shed light on the structure of the distance matrix built on the condition phase-space. The results of the community detection method applied to a compendium of 466 environmental conditions, both in the biomass-acetate and in the biomass-succinate space, are reported as Supplementary Information.

The importance of mapping microarray profiles to a metabolic model before clustering their associated conditions, compared to the clustering performed directly on gene expression data, is due to a number of reasons. First, multi-omic models provide means for clustering genes in their relative pathway; as a result, pathways can be effectively clustered and ranked through an effect-based approach (i.e., looking at the distance between their output outcomes in the phenotypic space) rather than looking merely at the expression profile of their genes. Second, the model acts as a ranking and noise-reduction tool, since the effect of low-importance genes is filtered out even if their expression is highly variable across conditions; without the multi-omic model, these genes would be incorrectly regarded as key genes to differentiate conditions. Third, performing inference directly on gene expression values may lead to incorrect prediction of the centrality of a gene whose level seems to be highly correlated with many other genes, but with only a marginal role in the metabolism (e.g., no impact on the biomass and on key metabolites).

The use of gene expression and codon usage as layers of the multi-omic model allows simulating growth on different media or environmental conditions. On the integration of gene expression data in the metabolic model, we refer the interested reader to the comprehensive review and evaluation by Machado and Herrgård^66^. We note that the vast majority of the related methods (e.g., mCADRE^67^, GIMME^68^, iMAT^69^) need binarization or discretization of expression values. Conversely, we share with E-Flux^70^ and PRIME^71^ the real-valued gene expression approach. As a result, we do not need to set any arbitrary threshold, and therefore we do not need to assume that only some reactions are present in the model. Threshold-based approaches are prone to error when changes in the gene expression level for different conditions are very small and therefore below the significance threshold; as a result, with a threshold-based method, these changes would not affect the present/absent calls of reactions, incorrectly predicting the absence of any effect on the metabolism. A further limitation of the related methods is that considering only one objective or a linear combination of objectives (usually encoded in the biomass reaction) is not sufficient to find all the possible metabolic states in which the maximization of a second product is performed concurrently with the maximization of the biomass on various substrates.

Importantly, in this study, the use of a multiobjective algorithm is key to obtain all the possible trade-off relationships among objectives, thus overcoming one of the major limitations of bilevel FBA, namely the estimation of only one point solution in the bidimensional objective space. With our multiobjective approach, we provide a wider range of solutions ensuring optimal values for all the objectives. Changing flux rates or performing gene over- and under-expression suggested by METRADE is less straightforward than performing gene knockout. However, several synthetic biology tools have been recently proposed to address this issue. For instance, the expression level can be regulated through ribosome binding site variants^59^, RNA programming devices^57^, and engineering of ligands to create sensors for regulation of gene expression^58^.

Additionally, our method shows how integrating multiple ‘omics can be used to compare different bacterial strains and evaluate the optimal behavior of a bacterium under various conditions. Specifically, the environmental conditions are mapped to genotypic data (expression profiles), and finally to phenotypic data (predictions of two or more optimized variables). This enables the use of the Pareto front, previously used only as an indication of where the metabolism operates^72^, and its hypervolume as indicators of the evolution of a strain. In the objective space, we showed how it is possible to further study this map through component analysis and spectral methods for community detection. A further extension of the framework could be inferring the topology of the network of conditions using a multidimensional scaling approach^73^. Such computational analysis can have a great impact especially for the large fraction of microorganisms that have been already identified but never cultured so far. For instance, a step forward would be to infer pathways to combat condition-dependent infections caused by bacteria involved in both plant and animal infections.

METRADE is made available as an extension of the COBRA 2.0 toolbox, and can be easily integrated with additional methods to investigate the Pareto front: (i) the *sensitivity analysis*, to quantify the importance of parameters and variables in the model; (ii) the *robustness analysis*, to evaluate how a proposed strain is robust to local and global perturbations^32^; (iii) the *identifiability analysis*, to find functional relations between variables^74^; and (iv) the *ε-dominance analysis*, to consider all the feasible solutions that are dominated with a tolerance *ε* with respect to Pareto solutions. The compendium of transcriptome analyses was mapped from the genotype to the phenotypic phase space in order to quantify the phenotypic changes in different growth conditions. As a result, we will be able to predict the phenotypic changes that occur after condition shifts and during the evolutionary adaptability.

Our software tool offers the opportunity to analyze an organism in a dynamic multi-omic fashion (genomics, transcriptomics, proteomics)^75^,^76^, e.g. by evaluating temporal changes in gene expression, codon usage and flux rates at various cellular levels. This allows for these layers to be interpreted as a whole, and to evaluate connections and interactions among them. Most phenotypic traits are controlled by many genes, but a global picture of the genotype-phenotype map is lacking; METRADE provides a starting point for modeling adaptive dynamics and for elucidating genotype-phenotype relationships. Further extensions may focus on combining estimated mutation rates, transcriptomic program complexity and variance, and selection coefficients, with the aim of providing an upper-bound estimation of the number of traits that can become and remain adapted by direct natural selection, i.e. the “many-traits” and “few-traits” phenotype-fitness maps^77^. The framework can also be extended to other bacteria that show variation of codon usage^5^, and to other environments (a very interesting medical application would be to simulate *in silico* the tumor conditions analyzed for the *Clostridium* bacteria^78^).

Finally, METRADE can be used to detect “internal” communities of conditions, where the closeness is measured on the response of the multi-omic model; it is therefore possible to create a correspondence with the “external” communities of conditions, where the closeness is usually knowledge-based or measured directly on the features of the environmental conditions. Due to the continuous nature of METRADE, we expect it will be used for calibration of genome-scale models, and in combination with dynamical aspects of FBA with the aim of detecting communities of conditions over time (e.g., reiterated shifts to completely different external conditions or growth environments). Coupled with advanced prediction techniques, for instance Bayesian “missing values” methods, METRADE can infer the response to conditions for which gene expression data are missing or incomplete. Therefore, it could represent an innovative tool for biologists for investigating important aspects of bacterial evolution, such as: (i) how genomic, metabolic, transcriptomic variations shape the complex adaptation landscape of bacteria; (ii) the ecological (condition-based) degree of coherency for bacterial genospecies; (iii) the relationship between speciation, ecotypes and ecological (condition-based) diversity; (iv) the adaptive response to different dosages of antibiotics and bacteriostatic chemicals.

## Methods

### Multiobjective optimization

When two or more tasks performed by a bacterial metabolic network are in conflict with each other, the Pareto front, obtained as a result of a multiobjective optimization routine, is a useful tool to seek trade-off solutions. In turn, the aim of multiobjective optimization in biological models is to optimize (maximize or minimize) the secretion or uptake of multiple target metabolites.

In a given multidimensional objective space, the set of points *f*(*x*) such that there does not exist any other point dominating *f*(*x*) at all tasks (objective functions) is called *Pareto front*. The points constituting the Pareto front are said to be *nondominated*. The remaining points, i.e. those associated with a point in the search space but not contained the Pareto front, are said to be *dominated*. More formally, let *f*_1_, ..., *f*_*r*_ be *r* objective functions to be maximized or minimized. The multiobjective optimization problem is the problem of optimizing the vector function *f*(*x*) = (*f*_1_(*x*),*f*_2_(*x*), ..., *f*_*r*_(*x*)), where *x* is the variable (vector) to be optimized in the search space. For the maximization (minimization) problem, this is achieved through the search for all the *Pareto optimal* vectors x^*^, namely all those x^*^ in the search space for which there does not exist any point *x* such that 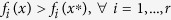 (or 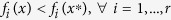 for the minimization problem). When the objective functions *f*_*i*_ in the organism are in conflict with each other, the term *optimizing* can be thought of as seeking trade-off solutions.

For the multiobjective optimization, METRADE includes a parallel genetic algorithm (PGA) inspired to NSGA-II^33^. Our PGA is parallel, easy to use, and suited for black-box analysis. For each generation of the algorithm, we provide the Pareto optimal solutions, in order to evaluate the evolution of the Pareto front. This loop is repeated until the solutions set does not improve, or until an individual with a desired phenotype is achieved. The number of generations and the cardinality of the population are parameters chosen by the user. In our experiments we consider 1500 populations of 1000 individuals each, in order to ensure an extensive exploration of the objective space. Each point *f*(x^*^) of the Pareto front is not merely a specific optimal model in the objective space, but also a gene expression array x^*^ representing a specific genotype in the variable search space. All the computations have been carried out on a machine with two 2.66 GHz 6-Core Intel Xeon processors and 64 GB of RAM.

We remark that this approach has advantages over reducing the multiobjective problem to a single-objective optimization. Summarizing two or more objectives in a single objective (e.g. through a weighted sum) brings in the problem of choosing the weights appropriately, and most importantly does not permit to recover non-convex sections of the Pareto front.

### The hypervolume indicator

The Pareto front is the set of all the nondominated points of the objective space. The dominated points are still part of the objective space, associated with a point in the search space, but they do not belong to the Pareto front. A measure of the volume of the dominated portion of the objective space, i.e. the part underlying the Pareto front, is the *hypervolume indicator*^51^. The hypervolume allows comparing different Pareto sets and evaluating the evolution of a Pareto set over time. In our two-objective spaces, we choose *O* = (0,0) as a reference point (since our aim is the maximization of the objectives), and therefore we define the hypervolume indicator of a subset 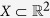, representing the Pareto front, as the Lebesgue measure of the space dominated by *X* with respect to the reference point *O*:


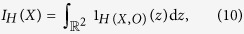


where *H* is the set of points dominated by the Pareto front *X* with reference point *O*; **1**_*H*(*X*,*O*)_ indicates the characteristic function and has value 1 at points of *H*(*X*,*O*) and 0 at points of 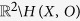. When the number of Pareto solutions is finite, i.e. *X* = {(*x*_1_, *y*_1_), …, (*x*_*n*_,*y*_*n*_)}, with the *x*_*i*_ sorted, the hypervolume equals


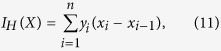


where for convenience of notation we set *x*_0_ = 0. Given the set of Pareto optimal solutions, the hypervolume can be exploited in the decision-making process, e.g. through the selection of a hypervolume-maximizing subset of the Pareto-optimal set.

### Spectral methods using the flow matrix for community detection

Let *G* = (*V*,*E*) be a graph with 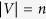, 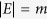. Formally, the flow matrix is a real valued matrix 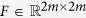 defined as


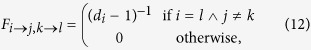


where *i*, *j*, *l*, *k* range over the nodes of the graph, and *d*_*i*_ is the degree of the *i*th node of the graph. Rows and columns of *F* are both associated with the *2m* edges of *G*. The flow matrix of *G* is a conservative-flow version of the non-backtracking matrix^79^, recently proposed for community detection, where the conservation of flow is achieved by normalizing all the matrix entries by the node degrees^52^.

Let us consider the case where the network is split into two communities of nodes, and let 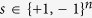 be the vector assigning each node to its community (+1 if assigned to the first, −1 if assigned to the second). The modularity *Q* of the flow matrix can be expressed using a scalar quadratic form as


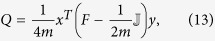


where 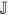 is the 2 *m* × 2 *m* all-ones matrix, 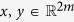 are two vectors defined such that the entry associated with each edge is equal to the group index (+1 or −1) of the node to which the edge is pointing^52^, namely 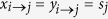.

Maximizing the modularity would yield the best separation of nodes into communities. In order to maximize the modularity over all the possible assignments *s* of nodes to communities, the condition that *x* and *y* contain only +1 and −1 can be relaxed. This allows them to take real values, but needs the constraint 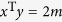 to limit *Q*. This is a constrained maximization problem and can be solved with the method of Lagrange’s undetermined multipliers. Combining (13) with the constraint 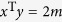 multiplied by the Lagrange multiplier *λ*, and differentiating with respect to *x* gives the condition of maximum modularity:


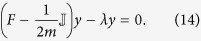


Therefore, a solution for the maximization problem is an eigenvector of 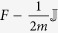 with eigenvalue *λ*. It follows that the maximum modularity is 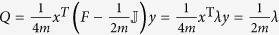, being 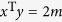. As a result, the solution of maximum modularity is the eigenvector associated with the leading eigenvalue of 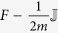.

By definition of 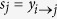, the number of elements of *y* associated with a node *j* is equal to its degree *d*_*j*_. In the relaxed version of the problem, we have therefore different elements 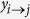 estimating the same attribute *s*_*j*_ of node *j*. We approximate a solution *s*_*j*_ for the original unrelaxed maximization problem as


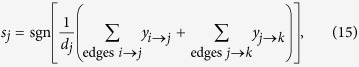


where 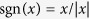 and sgn(0) = 0. Note that both *F* and 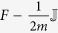 have the unit vector (normalized accordingly) as eigenvector. In *F*, the unit vector is an eigenvector with eigenvalue 1. Indeed, each *i* → *j* row has a 1/(*d*_*i*_ − 1) entry for every pair of edges *i* → *j*, *k* → *i* with 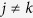 (*d*_*i*_ − 1 entries in total, since the case *j* = *k* is excluded), and therefore the sum of each *i* → *j* row is 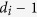 elements of type 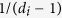. By the Perron-Frobenius theorem, the unit vector and 1 constitute the leading eigenvector/eigenvalue pair (see also Fig. 8). We finally compute the Fiedler eigenvector of *F*, i.e. the eigenvector associated with its second eigenvalue, because it equals the leading eigenvector of 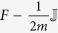, which we needed to maximize the modularity and classify the nodes into the communities. Indeed, 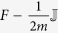 has the unit vector as eigenvector but with associated eigenvalue 0, while all the other eigenvectors and eigenvalues are the same^52^.

## Additional Information

**How to cite this article**: Angione, C. and Lió, P. Predictive analytics of environmental adaptability in multi-omic network models. *Sci. Rep.* **5**, 15147; doi: 10.1038/srep15147 (2015).

## Supplementary Material

Supplementary Information

Supplementary dataset 1

Supplementary dataset 2

Supplementary dataset 3

Supplementary Information

## Figures and Tables

**Figure 1:**
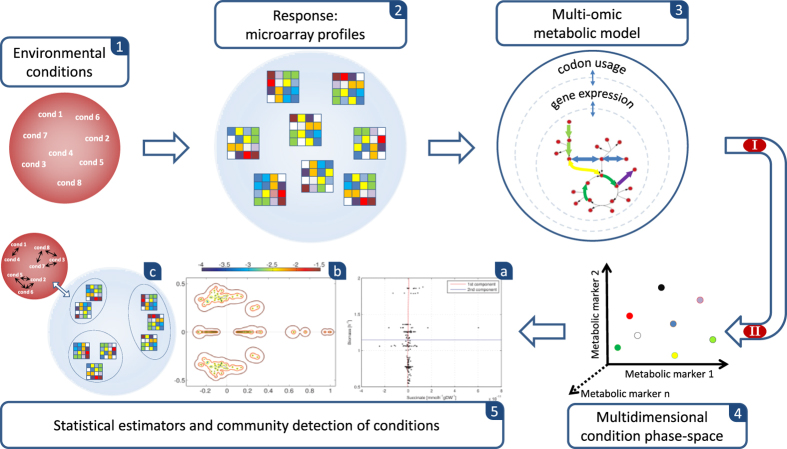
Pipeline of METRADE (MEtabolic and TRanscriptomics ADaptation Estimator). The idea behind the methods proposed in this study is that an accurate prediction on the relations among conditions should not disregard a multi-omic model to associate conditions to a phenotypic outcome in a set of objective spaces; indeed, a multi-omic inference cannot be performed by looking only at the gene expression profiles associated with the conditions, but requires a mapping to the phenotype that estimates the actual effect of each condition on the bacterial physiology. (See *Discussion* for further comments on the rationale behind METRADE.). **Part I (panels 1–3).** The response to environmental conditions (1) in which E. coli is grown (e.g., low or high glucose, aerobic or anaerobic, pH changes, antibiotics, heat shock) is measured through Affymetrix Antisense2 microarray expression profiling (2). To evaluate the environmental conditions and detect their community structure, we derive a multi-omic model (3) of the *E. coli* metabolism, taking into account gene expression and codon usage. **Part II (panels 4,5)**. (4) The model is able to account for multiple growth conditions and temporal multiobjective evolution towards the production of selected metabolites through the Pareto front. It is also able to associate each environmental condition with a single point in a multidimensional condition phase-space. The adaptability to one condition is given by the time evolution of the bacterial genome, which can be estimated by the hypervolume indicator. (5) We use a set of statistical estimators defined on our multi-omic model with the aim of analyzing the adaptability to experimental conditions. We apply the principal component analysis (5a) to the condition space in order to investigate the directions with largest variance, the pseudospectrum and its bagplot (5b) to shed light on the structure of a distance matrix built on the condition phase-space, and a spectral method for community detection to infer condition similarities (5c) according to the *E. coli* response in the multiobjective space.

**Table 1:** Values of *γ*, the principal component coefficients pc_1_ and pc_2_ (expressed as pair (*a*,*b*) of the line *ax* + *by* = 0), and the principal component variances *l*_1_ and *l*_2_ (i.e., the eigenvalues of the covariance matrix) of the conditions in the acetate-biomass (top) and succinate-biomass (bottom) objective spaces.

*γ*	pc_1_	pc_2_	*l*_1_	*l*_2_
Acetate-biomass				
1	(0.9997, 0.0247)	(0.0247, −0.9997)	12.9026	0.0460
100	(0.9997, 0.0251)	(0.0251, −0.9997)	12.7476	0.0463
500	(0.9996, 0.0299)	(0.0299, −0.9996)	9.5999	0.0462
1000	(0.9999, 0.0161)	(0.0161, −0.9999)	11.2712	0.0597
Succinate-biomass				
10^2^	(9.7072 · 10^−14^, −1)	(−1, −9.7072 · 10^−14^)	0.0542	4.1806 · 10^−25^
10^3^	(−8.6034 · 10^−13^, 1)	(−1, −8.6034 · 10^−13^)	0.0626	1.5392 · 10^−23^
10^4^	(1, −2.8217 · 10^−3^)	(−2.8217 · 10^−3^, −1)	2.1323	0.1267
10^5^	(−1, −3.2925 · 10^−4^)	(3.2925 · 10^−4^, −1)	37.6676	0.0121

The eigenvalues *l*_1_ and *l*_2_ indicate the variance explained by the first and second principal components respectively. The eigenvector with the largest eigenvalue *l*_1_ represents the direction of maximal variation of the points in the objective space.
