# Erratum: Predictive analytics of environmental adaptability in multi-omic network models

**DOI:** 10.1038/srep26266

**Published:** 2016-05-20

**Authors:** Claudio Angione, Pietro Lió

Scientific Reports 5: Article number: 15147; 10.1038/srep15147 published online: 10202015; updated: 05202016.

This Article contains typographical errors.

In the Results section under subheading ‘METRADE: a novel method to integrate and optimize gene expression and codon usage in FBA’,

“Through the function *h*(.), the gene expression arrays have a continuous effect on the FBA model, rather than only an on/off effect on reactions as in the Boolean approaches.”

should read:

“Through the function *h*(^·^), the gene expression arrays have a continuous effect on the FBA model, rather than only an on/off effect on reactions as in the Boolean approaches.”

In the Results section under subheading ‘Mapping genotype-phenotype associations to multidimensional objective spaces’,

“Then, we solve the bilevel problem (4) replacing the function *h* with the function 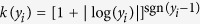, where 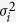 is the variance of the gene set responsible for the *i*th reaction, and *γ* is a weight for the variance.”

should read:

“Then, we solve the bilevel problem (4) replacing the function *h* with the function 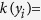 
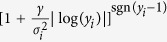
, where 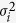 is the variance of the gene set responsible for the *i*th reaction, and *γ* is a weight for the variance.”

