# Supplementary material for: Predictive analytics of environmental adaptability in multi-omic network models
Source: Sci Rep. 2015 Oct 20;5:15147. doi: 10.1038/srep15147 (PMC4611489; doi:10.1038/srep15147)
Supplement: Supplementary Information [file srep15147-s1.pdf]

Predictive analytics of environmental adaptability in multi-omic network models

Claudio Angione[1,*] **and Pietro Lió**[1]

[1]Computer Laboratory - University of Cambridge, UK
[*]claudio.angione@cl.cam.ac.uk

ABSTRACT

Bacterial phenotypic traits and lifestyles in response to diverse environmental conditions depend on changes in the internal molecular environment. However, predicting bacterial adaptability is still difficult outside of laboratory controlled conditions. Many molecular levels can contribute to the adaptation to a changing environment: pathway structure, codon usage, metabolism. To measure adaptability to changing environmental conditions and over time, we develop a multi-omic model of *Escherichia coli* that accounts for metabolism, gene expression and codon usage at both transcription and translation levels. After the integration of multiple omics into the model, we propose a multiobjective optimization algorithm to find the allowable and optimal metabolic phenotypes through concurrent maximization or minimization of multiple metabolic markers. In the condition space, we propose Pareto hypervolume and spectral analysis as estimators of short term multi-omic (transcriptomic and metabolic) evolution, thus enabling comparative analysis of metabolic conditions. We therefore compare, evaluate and cluster different experimental conditions, models and bacterial strains according to their metabolic response in a multidimensional objective space, rather than in the original space of microarray data. We finally validate our methods on a phenomics dataset of growth conditions. Our framework, named METRADE, is freely available as a MATLAB toolbox.

Supplementary material

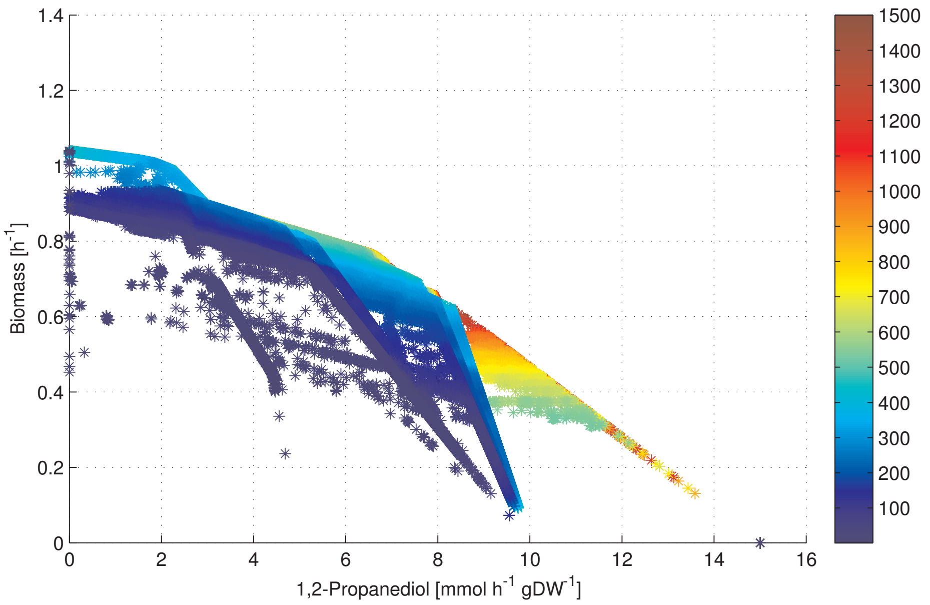

Figure S1. Pareto front produced by METRADE when maximizing 1,2-propanediol and biomass production in anaerobic conditions. Solutions are denoted by progressively warmer colors according to the time step of the PGA in which they have been generated adaptively.

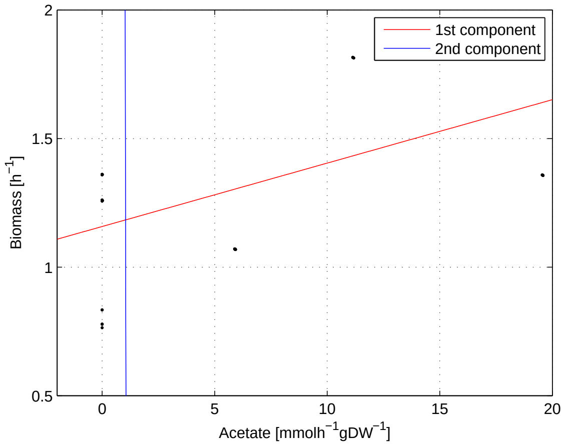

(a) $\gamma = 1$

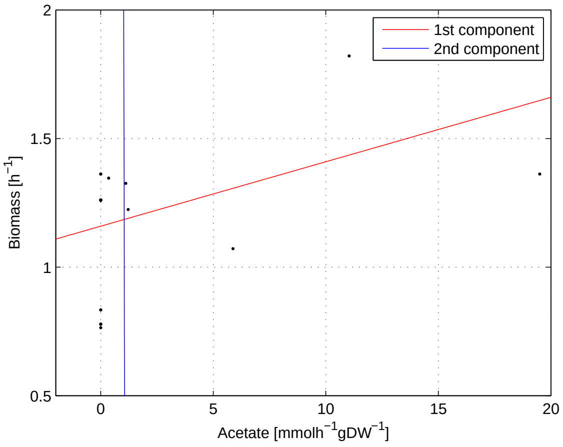

(b) $\gamma = 100$

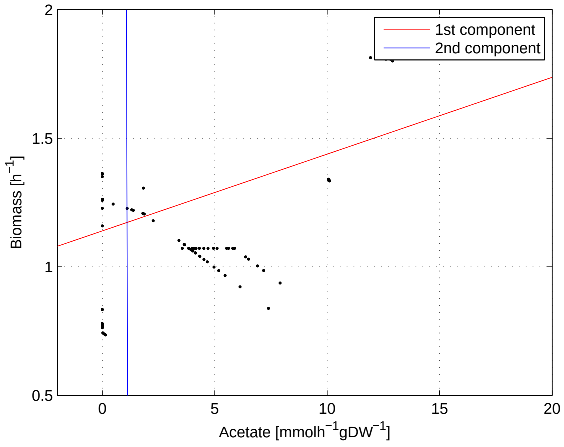

(c) $\gamma = 500$

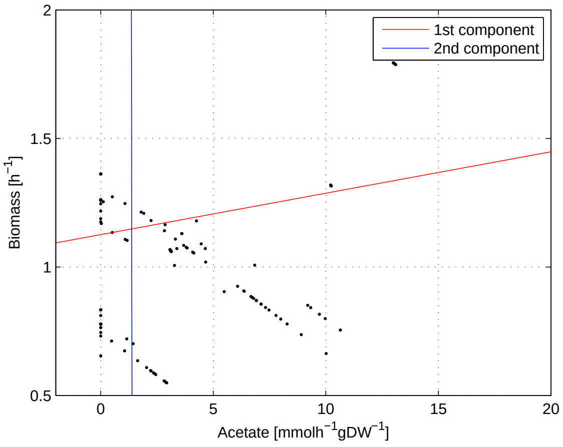

(d) $\gamma = 1000$

Figure S2. PCA in the acetate-biomass objective space. The two principal components are computed using the principal component analysis on the centered data $(x - \mu_x, y - \mu_y)$, but are plotted on the original data. The slope of the first principal component (i.e., the direction of maximum variance of the data), depends on the parameter γ, the multiplicative factor that influences the effect of the gene expression data on the upper and lower bounds of the reaction fluxes in the FBA model. The second principal component is always perpendicular to the first component (not highlighted in these plots due to different scales used).

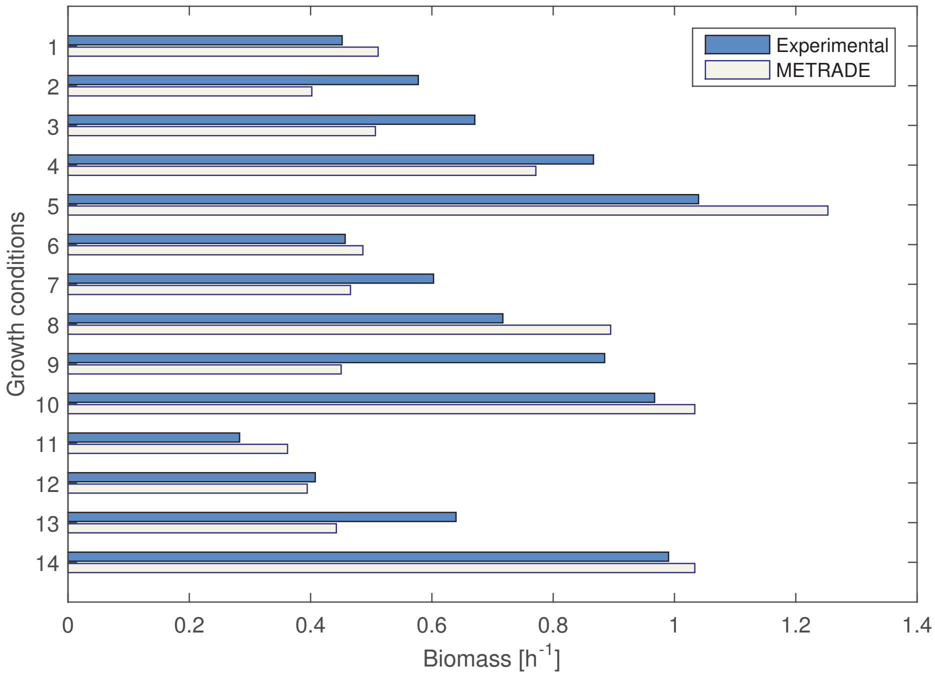

Figure S3. Comparison between the measured growth rates and those predicted by METRADE in 14 growth conditions. See additional Supplementary files for details on each condition.

Supporting Information Legends

Additional file 1 — all_METRADE_points.xlsx

Feasible (nondominated and dominated) solutions found by METRADE for the acetate-biomass and succinate-biomass optimization problems, with and without noise added to the initial step of the optimization of the gene expression profiles. For each solution, we report the amount of natural (biomass) and second (acetate or succinate) objectives, the rank (0 if dominated, -1 if nondominated), the index of the solution and the population in which it has been generated by the PGA. Each row represents a solution in the bi-objective space, and is associated with a specific gene expression profile found by the algorithm in the search space.

Additional file 2 — data_only_proteomics.xlsx

Dataset used in this study to validate METRADE. We include the expression levels by in 14 conditions. We indicate average expression level and variance for each gene and for each condition. For each condition, we report the experimentally measured growth rate and the biomass predicted by METRADE.

Additional file 3 — communities_conditions.xlsx

Community detection for experimental conditions based on the outcome in the acetate-biomass and succinate-biomass objective spaces.

Additional file 4 — METRADE.zip

METRADE source code.
