# Supplementary material for: Predictive analytics of environmental adaptability in multi-omic network models
Source: Sci Rep. 2015 Oct 20;5:15147. doi: 10.1038/srep15147 (PMC4611489; doi:10.1038/srep15147)
Supplement: Supplementary Information [file srep15147-s5.zip › source code METRADE/5) pseudospectra/eigtoollib/eigtool_docs.html]

Redirect to EigTool Documentation

You should be automatically forwarded to the EigTool documentation website. If this does not
happen click html/eigtool/documentation/index.html.
