# Supplementary material for: Predictive analytics of environmental adaptability in multi-omic network models
Source: Sci Rep. 2015 Oct 20;5:15147. doi: 10.1038/srep15147 (PMC4611489; doi:10.1038/srep15147)
Supplement: Supplementary Information [file srep15147-s5.zip › source code METRADE/5) pseudospectra/eigtoollib/html/eigtool/documentation/buttons/index.html]

EigTool Buttons


# EigTool Buttons

If a menu item in the table below is a hyperlink, click to see
examples.

|  |  |
| --- | --- |
| Go!/Stop! | This button will only be enabled if options have been changed that require recomputation of the pseudospectra. When it is pressed, it will change to a red Stop! button that can be used to stop the computation in progress. |
| Pause/Resume | This button is only enabled when a computation is in progress. If it is pressed, the computation will be paused until the button is pressed again. |
| Field of Vals. | This button will compute and display the field of values (numerical range) of the matrix. If you wish to change the number of points used in the compution of the field of values, use the menu option Accuracy of FoV Comp. in the Extras menu. To remove the field of values from the plot, simply click on the button again. If you wish to turn it on again, it will not need to be recomputed unless the number of points defining it has been changed. |
| Mode + Cond. No. | After clicking on this button, you will be asked to select an eigenvalue by clicking on the pseudospectra plot; the eigenvalue nearest to your click will be chosen. A figure will then be produced that plots the eigenmode corresponding to this eigenvalue against its index. The top plot shows an envelope produced by plotting the absolute value and minus the absolute value of the eigenmode, and the real part will be shown as a cyan line. The bottom plot shows the absolute value of the eigenmode on a log scale, useful for seeing how quickly an eigenmode is decaying. In addition to the plot of the eigenmode, the condition number of the eigenvalue is computed and shown in the top plot. Note that a large condition number indicates that the eigenvalue is very sensitive to perturbations. If a second eigenmode is plotted, it will overwrite the first one unless the Keep this Eigenmode option is selected from the Eigenmode Controls menu of the eigenmode figure. If it is, a new figure will be created for the second (and subsequent) eigenmodes. |
| Pmode + epsilon | This button produces a plot that is similar to the Mode + Cond. No. plot, except that this time any point in the complex plane can be selected, and a pseudoeigenmode corresponding to this point is drawn. This time, the value of (the inverse of) the resolvent norm at the point is shown in place of the eigenvalue condition number. The real part of a pseudomode is plotted in magenta to distinguish it from an eigenmode. The pseudomode shown is the right singular vector corresponding to the smallest singular value of the matrix zI-T, where z is the point selected by the user. Pseudomodes appear in separate figures from eigenmodes, but as with eigenmodes, a second pseudomode will use the same figure unless the Keep this Psuedomode option is selected. |
| 3D Plot | This button creates a new figure with a 3D surface plot of the current pseudospectra data. An additional menu, `Plot Controls' is added to this figure, allowing control over the look of the plot. |
| Quit | Exit EigTool. |

# Some specific examples

## Field of Vals.

This example is based on the Kahan matrix in the Demos menu; the field of values is shown as a
dashed black line:

## Mode + Cond. No.

The figure below shows the eigenvalues and pseudospectra of Davies'
matrix, with the cyan circle indicating the eigenvalue selected after
clicking on Mode + Cond. No:

The corresponding eigenmode with its condition number is shown in this figure:

## Pmode + epsilon

Using the same matrix as for Mode + Cond. No.,
we can plot a pseudomode corresponding to z=15+10i:

## 3D Plot

Using the built in `Companion' matrix demo, clicking on 3D plot
creates the following figure:

---

EigTool home page.
