# Supplementary material for: Predictive analytics of environmental adaptability in multi-omic network models
Source: Sci Rep. 2015 Oct 20;5:15147. doi: 10.1038/srep15147 (PMC4611489; doi:10.1038/srep15147)
Supplement: Supplementary Information [file srep15147-s5.zip › source code METRADE/5) pseudospectra/eigtoollib/html/eigtool/documentation/contents.html]

Contents of EigTool Documentation


# Contents

EigTool Documentation

- Quick start
  - Matrix Types
    - Dense Square Matrices- Sparse Square Matrices- Dense Rectangular Matrices- Interactive
    EigTool use
    - Menus
      - File
        - New Matrix- Save Current State- Save Current State As...- Export: Eigenvalues- Export: Original Matrix- Export: Pseudospectra Data- Export: Schur Factor- Printable Plot...- Quit- Extras
          - Options Code for Printing- Save for Quick Print Creation- Projection Level- Colour- Thick Lines- Arbitrary Levels- Accuracy of FoV Comp.- Display Eigenvalues- Display Pseudospectra- Display Dimension- Display Gridpoints- Display Imaginary Axis- Display Unit Circle- Display Colourbar- ARPACK/eigs
            - Maximum Subspace Size (p)...- Convergence Tolerance (tol)...- Maximum Number of Restarts (maxit)...- Starting Vector (v0)...- Automatic Axes- Show Progress...Ritz Values/Shifts- Show Progress...All Shifts- Show Progress...Pseudospectra- Transients
              - Matrix Powers- Matrix Exponentials- Compute a Bound- Best Estimate Lower Bound- Information- Numbers
                - Eigenvector Matrix Cond. No.- Departure from Normality- Numerical Abscissa- Pseudospectral Abscissa- Pseudospectral Radius- Spectral Abscissa- Spectral Radius- Display Points- Demos
                  - Finer grid (slower)- Dense matrices- Sparse matrices- Help
                    - EigTool Documentation- Pseudospectra Tutorial- EigTool Homepage- Pseudospectra Gateway- Buttons
        - Go!/Stop!- Pause/Resume- Field of Vals.- Mode + Cond. No.- Pmode + epsilon- 3D Plot- Quit- Panes
          - Figure Axes- Direct/Iterative- Contour Levels- Mesh- Using EigTool from the command line
      - Command Line Options
        - Basic EigTool Operation- Using OPTIONS to Control the Plot- Advanced Calling Sequences- Changing the default options
        - EigTool Preferences
          - Setting a preference- Resetting all the preferences
