# Supplementary material for: Predictive analytics of environmental adaptability in multi-omic network models
Source: Sci Rep. 2015 Oct 20;5:15147. doi: 10.1038/srep15147 (PMC4611489; doi:10.1038/srep15147)
Supplement: Supplementary Information [file srep15147-s5.zip › source code METRADE/5) pseudospectra/eigtoollib/html/eigtool/documentation/general.html]

EigTool Documentation


EigTool is a MATLAB GUI for computing eigenvalues, pseudospectra
and related quantities for nonsymmetric matrices, both dense and
sparse. The following list summarises the main features:

- Graphical interface to eigs- Computation of eigenvalue condition numbers- Plots of eigenmodes- Computation of the field of values (numerical range)- Computation of pseudospectra

This documentation assumes that you have already downloaded the latest version of EigTool, and
have followed all of the installation instructions on that page.

### Quick start

The simplest way to begin using EigTool is to type eigtool at
the MATLAB command prompt; EigTool will start up and wait for you to
give it a matrix:

Alternatively, if you already have a matrix you would like to
compute the pseudospectra of (say A), type
eigtool(A).

If you are unfamiliar with pseudospectra, try running the
Pseudospectra Tutorial, available through the Help menu, or there are
many examples that can be viewed using the Demos menu. For a more
detailed look, the  contains many more examples
and explinations to help start you off. If you already have a matrix,
select the New Matrix menu item to
start exploring. As a rule, EigTool computations are usually quite
fast for matrices of dimensions in the low hundreds.

For a description of all of the types of matrices that EigTool can
handle, see Matrix Types.

### Interactive EigTool use

Once the eigenvalues and pseudospectra of your matrix have been
computed, the full list of features of EigTool can be used:

These break down into three categories:

- Menus- Buttons- Panes

### Using EigTool from the command line

In addition to its interactive user interface, EigTool can be called
from the command line with options to control the look of the
plot. Full details are given in Command Line Options.

### Changing the default options

All of EigTool's default options can be modified using MATLAB
preferences. For example, the default behaviour of EigTool displays
the matrix dimension on the plot unless this is overridden using command line options; some users may
prefer that the dimension is not displayed by default. Full details
are given in EigTool Preferences.

---

The original version of EigTool was designed and built at Oxford University
during 2000-2002 by Thomas G. Wright in collaboration with Mark Embree
and Lloyd N. Trefethen. The present version is maintained by Mark Embree;
bug reports should be directed to him at
embree@rice.edu.

---

### Disclaimer

This software package is delivered "as is". The author makes no
representation or warranties, express or implied, with respect to the
software package. In no event shall the author be liable for loss of
profits, loss of savings, or direct, indirect, special, consequential,
or incidental damages.

---

EigTool home page.
