# Supplementary material for: Predictive analytics of environmental adaptability in multi-omic network models
Source: Sci Rep. 2015 Oct 20;5:15147. doi: 10.1038/srep15147 (PMC4611489; doi:10.1038/srep15147)
Supplement: Supplementary Information [file srep15147-s5.zip › source code METRADE/5) pseudospectra/eigtoollib/html/eigtool/documentation/matrixtypes.html]

Matrix Types


# Matrix Types

It is possible to call EigTool with three different types of matrices:
Dense Square Matrices, Sparse
Square Matrices and Dense Rectangular
Matrices.

## Dense Square Matrices

If a dense square matrix has dimension smaller than about 1000, it is
possible to compute a Schur decomposition of the matrix in a
reasonable amount of time. For matrices such as these, this is the
first thing EigTool does, obtaining the eigenvalues from the diagonal
entries of the triangular Schur factor. Assuming that no options have been defined, the axes
to compute the initial pseudospectra on are dertermined by the
position of the eigenvalues. A default number of grid points based on
the matrix dimension will be chosen, and once the singular value data
has been computed appropriate Contour Levels are calculated. As
an example, the command eigtool(gallery('grcar',100))
produces the figure:

If the matrix is larger than 1000 then the Schur decomposition can
be prohibitively expensive. In this case, EigTool offers the user the
choice of using iterative
methods instead (ARPACK/eigs) to compute a few eigenvalues of
interest. As an example, consider eigtool(triu(randn(1001),-1)):
EigTool asks whether you would like to use iterative methods:

If we click on ARPACK/eigs, and ask for 20 eigenvalues of largest
modulus, EigTool fairly quickly produces the following plot, which is
obtained by projecting the original matrix onto a lower dimensional
invariant subspace, and approximating the pseudospectra with the
pseudspectra of this projected matrix:

## Sparse Square Matrices

The case of sparse, square matrices is a little more complicated. If
the matrix is small (dimension less than 1000), you will be offered
the chance to convert it to a dense matrix, and the proceed using the
techniques described above. Assuming that this is
not done, there are two possibilities: you can use iterative methods (ARPACK/eigs) to
compute some eigenvalues and approximate the pseudospectra locally, or
you can use a direct method to compute the (exact) pseudospectra only
in a user-specified region. This is particularly useful when trying to
see how well the approximate pseudospectra match the true
ones. As an example, we use the matrix pde225 from Matrix
Market: cd eigtool/private/; eigtool(mmread('pde225.mtx'));

Using ARPACK/eigs, looking for 20 eigenvalues
of largest modulus, we get the following eigenvalues and approximate
pseudospectra:

Repeating the
above command, but selecting Sparse Direct gives us the exact pseudospectra
(but no eigenvalues):

Note that
when using a sparse direct method, you will be prompted for the axes
to compute on unless you provide eigenvalues using the options.

## Dense Rectangular Matrices

The final class of matrices that EigTool can handle are those that are
dense and rectangular. The most common need for this kind of matrix is
the rectangular (n+1)-by-n Hessenberg matrix created by eigs,
whose pseudospectra are subsets of the pseudospectra of the original
matrix. EigTool can handle general rectangular matrices, though. Since
rectangular matrices have no eigenvalues in general, EigTool will
prompt for axes to compute the pseudospectra over, before behaving in
the usual way.

---

EigTool home page.
