# Supplementary material for: Predictive analytics of environmental adaptability in multi-omic network models
Source: Sci Rep. 2015 Oct 20;5:15147. doi: 10.1038/srep15147 (PMC4611489; doi:10.1038/srep15147)
Supplement: Supplementary Information [file srep15147-s5.zip › source code METRADE/5) pseudospectra/eigtoollib/html/eigtool/documentation/menus/airy_demo.html]

EigTool Airy Demo


# EigTool Airy Demo

Our "Airy operator" is an operator acting on functions of x
defined on [-1,1] according to the formula
where epsilon is a small parameter. The spectrum of this operator
is an unbounded discrete set contained in the half-strip Re z < 0,
-1 < Im z < 1. The pseudospectra approximately fill the whole
strip. The pseudospectra of this operator were first considered
by Reddy, Schmid and Henningson as a model of the more complicated
Orr-Sommerfeld problem [1]. Our M-file is based on a Chebyshev
collocation spectral discretization with epsilon = 4e-3.

[1]: S. C. Reddy, P. J. Schmidt and D. S. Henningson,
"Pseudospectra of the Orr-Sommerfeld operator",
SIAM J. Appl. Math. 53(1), pp. 15-47, 1993.

---

EigTool EigTool Demos Menu.
