# Supplementary material for: Predictive analytics of environmental adaptability in multi-omic network models
Source: Sci Rep. 2015 Oct 20;5:15147. doi: 10.1038/srep15147 (PMC4611489; doi:10.1038/srep15147)
Supplement: Supplementary Information [file srep15147-s5.zip › source code METRADE/5) pseudospectra/eigtoollib/html/eigtool/documentation/menus/arpack.html]

EigTool ARPACK/eigs Menu


# ARPACK/eigs Menu

Note that this menu will only be enabled when the ARPACK/eigs option
is selected in the Direct/Iterative pane. ARPACK is a
Fortran code for computing eigenvalues of large, sparse matrices;
eigs in MATLAB calls the Fortran ARPACK routines directly.

If a menu item in the table below is a hyperlink, click to see
examples.

|  |  |  |
| --- | --- | --- |
| Maximum Subspace Size (p)... |  | Select the maximum size that eigs will build a subpace out to (opts.p). |
| Convergence Tolerance (tol)... |  | Set the convergence tolerance for eigs (opts.tol) |
| Maximum Number of Restarts (maxit)... |  | Set the maximum number of restarts to take before quitting (opts.maxit) |
| Starting Vector (v0)... |  | Set the starting vector for eigs to use; you will be prompted to enter a MATLAB command to create the vector (opts.v0) |
| Automatic Axes |  | If this item is toggled on, the axes will automatically adjust during the eigs computation to ensure that all the Ritz values and shifts are visible. Once the axes are changed manually, this option is automatically deselected; to use automatic axes again, you need to select this menu item. |
| Show Progress... | Ritz Values/Shifts | Display the eigenvalues of the current projected matrix H at each implicit restart. Those being used as shifts will be drawn as red pluses, with those Ritz values that approximate the desired eigenvalues (not used as shifts) plotted as black dots |
|  | All Shifts | Same as above, but all of the shifts used so far will be plotted. |
|  | Pseudospectra | Compute the pseudospectra of the rectangular Hessenberg matrix at each implicit restart; this can give some idea of the nonnormality of the problem as the computation proceeds. Note that using this option will slow down the computation, especially when the original matrix is small. |

# Some specific examples

## Show Progress...Ritz Values/Shifts

This is the default option when computing eigenvalues using
ARPACK/eigs. Below we see the Elliptic PDE Demo after 6 implicit restarts: the eigenvalues
of the projected matrix being shifted with are shown as red plusses,
with those being kept as black dots.

## Show Progress...All Shifts

It can often be useful to see not only the shifts being used at the
current implicit restart, but all those that have been used
already. In the figure below, we see all the shifts used up to (and
including) the 8th implicit restart:

## Show Progress...Pseudospectra

The final option for viewing progress during an eigs computation is to
see the pseudospectra of the rectangular Hessenberg matrix after each
implicit restart, which gives some idea of the nonnormality of the
problem as the iteration progresses. The figure below shows the
pseudospectra of the Hessenberg matrix after the 9th implicit restart:

---

EigTool home page.
