# Supplementary material for: Predictive analytics of environmental adaptability in multi-omic network models
Source: Sci Rep. 2015 Oct 20;5:15147. doi: 10.1038/srep15147 (PMC4611489; doi:10.1038/srep15147)
Supplement: Supplementary Information [file srep15147-s5.zip › source code METRADE/5) pseudospectra/eigtoollib/html/eigtool/documentation/menus/basor_demo.html]

EigTool Basor-Morrison Demo


# EigTool Basor-Morrison Demo

This is a Toeplitz matrix of dimension N with a piecewise
continuous symbol. For N sufficiently large, each of the
illustrated pseudospectra will include the spectrum of the
infinite dimensional operator, but for this class of piecewise
continuous symbols, this value of N needs to be especially
large. The eigenvalues of this example have been studied by
Basor and Morrison [1] and the pseudospectra are illustrated
in [2].

[1]: Basor and Morrison "The Fisher-Hartwig conjecture and
Toeplitz eigenvalues", Linear Algebra Appl. 202 (1994),
pp. 129-142).

[2]: A. Boettcher, M. Embree, and L. N. Trefethen "Piecewise
continuous Toeplitz matrices and operators: slow approach
to infinity", Oxford University Computing Laboratory
Numerical Analysis Technical Report 00/19, August 2000.

---

EigTool EigTool Demos Menu.
