# Supplementary material for: Predictive analytics of environmental adaptability in multi-omic network models
Source: Sci Rep. 2015 Oct 20;5:15147. doi: 10.1038/srep15147 (PMC4611489; doi:10.1038/srep15147)
Supplement: Supplementary Information [file srep15147-s5.zip › source code METRADE/5) pseudospectra/eigtoollib/html/eigtool/documentation/menus/boeing_demo.html]

EigTool Boeing 767 Demo


# EigTool Boeing 767 Demo

These matrices of dimension 55 come from a flutter analysis of the
Boeing 767 aircraft reported in the SLICOT Control and Systems Library
(http://www.win.tue.nl/wgs/slicot.html).
This model has some adjustable
parameters. The unstable matrix (below) corresponds to a set of parameters
that lead to an unstable pair of eigenvalues slightly inside the
right half-plane. The stable matrix (bottom) corresponds to a different set of
parameters, found by Michael Overton, that lead to all eigenvalues in
the left half-plane.

---

EigTool EigTool Demos Menu.
