# Supplementary material for: Predictive analytics of environmental adaptability in multi-omic network models
Source: Sci Rep. 2015 Oct 20;5:15147. doi: 10.1038/srep15147 (PMC4611489; doi:10.1038/srep15147)
Supplement: Supplementary Information [file srep15147-s5.zip › source code METRADE/5) pseudospectra/eigtoollib/html/eigtool/documentation/menus/chebspec_demo.html]

EigTool Chebspec Demo


# EigTool Chebspec Demo

This matrix is the Chebyshev spectral differentiation matrix of
dimension N [1] of the first derivative operator d/dx
on [-1,1] with boundary condition u(1)=0.
The pseudospectra of the infinite dimensional operator
consist of half-planes, which is suggested in this
low-dimensional discretization by the straight lines
that form the rightmost part of each pseudospectral
boundary [2].

[1]: L. N. Trefethen, "Spectral methods in MATLAB",
SIAM, 2000.

[2]: L. N. Trefethen, "Psuedospectra of matrices", in
"Numerical Analysis 1991" (Dundee 1991), Longman Sci.
Tech., Harlow, 1992, 234-266.

---

EigTool EigTool Demos Menu.
