# Supplementary material for: Predictive analytics of environmental adaptability in multi-omic network models
Source: Sci Rep. 2015 Oct 20;5:15147. doi: 10.1038/srep15147 (PMC4611489; doi:10.1038/srep15147)
Supplement: Supplementary Information [file srep15147-s5.zip › source code METRADE/5) pseudospectra/eigtoollib/html/eigtool/documentation/menus/companion_demo.html]

EigTool Companion Demo


# EigTool Companion Demo

This matrix was suggested by Cleve Moler. It is the
companion matrix for the truncated power series of
exp(z). Note that serious rounding errors for N much
larger than 15 due to the large size of some entries.

---

EigTool EigTool Demos Menu.
