# Supplementary material for: Predictive analytics of environmental adaptability in multi-omic network models
Source: Sci Rep. 2015 Oct 20;5:15147. doi: 10.1038/srep15147 (PMC4611489; doi:10.1038/srep15147)
Supplement: Supplementary Information [file srep15147-s5.zip › source code METRADE/5) pseudospectra/eigtoollib/html/eigtool/documentation/menus/convdiff_demo.html]

EigTool Convection-Diffusion Demo


# EigTool Convection-Diffusion Demo

This is the 1-D convection-diffusion operator eu'' + u' on [0,1] with
u(0)=u(1)=0 for e=1/30, discretised using a Chebyshev spectral
method [1]. The boundaries of pseudospectra of the infinite
dimensional operator are shaped approximately like parabolas,
as already revealed on the right part of this low-dimensional
approximation [2].

[1]: L. N. Trefethen, "Spectral methods in MATLAB",
SIAM, 2000.

[2]: S. C. Reddy and L. N. Trefethen, "Pseudospectra of the
convection-diffusion operator", SIAM J. Appl. Math. 54(6),
pp. 1634-1649, 1994.

---

EigTool EigTool Demos Menu.
