# Supplementary material for: Predictive analytics of environmental adaptability in multi-omic network models
Source: Sci Rep. 2015 Oct 20;5:15147. doi: 10.1038/srep15147 (PMC4611489; doi:10.1038/srep15147)
Supplement: Supplementary Information [file srep15147-s5.zip › source code METRADE/5) pseudospectra/eigtoollib/html/eigtool/documentation/menus/convdiff_fd_demo.html]

EigTool Convection-Diffusion (FD) Demo


# EigTool Convection-Diffusion (FD) Demo

This is the 2-D convection-diffusion operator

-e\*laplacian(u) + u\_y

on the unit square with Dirichlet boundary conditions. The
discretisation is done using finite differences (see, e.g., [1]),
resulting in large, sparse matrices. The code is based on a
routine by Daniel Loghin.

[1]: L. Hemmingsson, "A Semi-circulant Preconditioner for the
Convection-Diffusion Equation", Numer. Math 81, 1998, 211-248

---

EigTool EigTool Demos Menu.
