# Supplementary material for: Predictive analytics of environmental adaptability in multi-omic network models
Source: Sci Rep. 2015 Oct 20;5:15147. doi: 10.1038/srep15147 (PMC4611489; doi:10.1038/srep15147)
Supplement: Supplementary Information [file srep15147-s5.zip › source code METRADE/5) pseudospectra/eigtoollib/html/eigtool/documentation/menus/davies_demo.html]

EigTool Davies Demo


# EigTool Davies Demo

This is an anharmonic oscillator, -u\_xx + cx2u, for c=1+3i, discretised
using a Chebyshev spectral method [3]. Since this discretization
includes numerous spurious eigenvalues, the matrix is projected
onto an appropriate invariant subspace to accelerate computation
of the pseudospectra. This example was first studied by Brian
Davies [1,2].

[1]: E. B. Davies, "Pseudo-spectra, the harmonic oscillator and
complex resonances", Proc. Roy. Soc. Lond. Ser. A 455 (1999),
pp. 585-599.

[2]: E. B. Davies, "Semi-classical states for non-self-adjoint
Schroedinger operators", Commun. Math. Phys. 200 (1999),
pp. 35-41.

[3]: L. N. Trefethen, "Spectral methods in MATLAB",
SIAM, 2000.

---

EigTool EigTool Demos Menu.
