# Supplementary material for: Predictive analytics of environmental adaptability in multi-omic network models
Source: Sci Rep. 2015 Oct 20;5:15147. doi: 10.1038/srep15147 (PMC4611489; doi:10.1038/srep15147)
Supplement: Supplementary Information [file srep15147-s5.zip › source code METRADE/5) pseudospectra/eigtoollib/html/eigtool/documentation/menus/demmel_demo.html]

EigTool Demmel Demo


# EigTool Demmel Demo

This is Demmel's matrix, which was perhaps the first matrix
whose pseudospectra (with N=3) appeared in a published
paper [1]. Demmel devised the matrix in order to disprove
a conjecture of Van Loan's [2].

[1]: J. W. Demmel, "A counterexample for two conjectures
about stability", IEEE Trans. Auto. Control, AC-32,
pp. 340-343, 1987.

[2]: C. Van Loan, "How near is a stable matrix to an
unstable matrix?", in R. Brualdi, et al., eds,
Contemporary Mathematics 47, Amer. Math. Soc., 1985

---

EigTool EigTool Demos Menu.
