# Supplementary material for: Predictive analytics of environmental adaptability in multi-omic network models
Source: Sci Rep. 2015 Oct 20;5:15147. doi: 10.1038/srep15147 (PMC4611489; doi:10.1038/srep15147)
Supplement: Supplementary Information [file srep15147-s5.zip › source code METRADE/5) pseudospectra/eigtoollib/html/eigtool/documentation/menus/demos.html]

EigTool Demos Menu


# Demos Menu

|  |  |
| --- | --- |
| Finer grid (slower) | Toggle this item to compute all subsequent demos using a finer grid, resulting in a plot with smoother contour lines. The computation will of course take longer. |
| Dense matrices | This sub-menu contains all of EigTool's dense matrix demos. For details of the individual demos, see Dense Demos. |
| Sparse matrices | This sub-menu contains all of EigTool's sparse matrix demos. For details of the individual demos, see Sparse Demos. |

## Dense Demos

Click on a thumbnail in the table below to see a full description for
that demo.

|  |  |
| --- | --- |
| Airy | Basor-Morrison |
| Boeing 767 | Chebspec |
| Companion | Convection-Diffusion |
| Davies | Demmel |
| Frank | Gallery 3 |
| Gallery 5 | Gauss-Seidel |
| Godunov | Grcar |
| Hatano-Nelson | Kahan |
| Landau | Orr-Sommerfeld |
| Random | Random Triangular |
| Riffle Shuffle | Transient |
| Twisted Toeplitz | |

## Sparse Demos

Click on a thumbnail in the table below to see a full description for
that demo.

|  |  |
| --- | --- |
| DWAVE | Convection-Diffusion |
| Elliptic PDE Markov Process | |  |
| Olmstead | RDB 800 |
| Sparse Random | Skew Laplacian |
| SUPG | Tolosa |

---

EigTool home page.
