# Supplementary material for: Predictive analytics of environmental adaptability in multi-omic network models
Source: Sci Rep. 2015 Oct 20;5:15147. doi: 10.1038/srep15147 (PMC4611489; doi:10.1038/srep15147)
Supplement: Supplementary Information [file srep15147-s5.zip › source code METRADE/5) pseudospectra/eigtoollib/html/eigtool/documentation/menus/extras.html]

EigTool Extras Menu


# Extras Menu

If a menu item in the table below is a hyperlink, click to see
examples.

|  |  |
| --- | --- |
| Options Code for Printing | Selecting this item outputs text to the MATLAB window which can then be cut and paste into a script. The text contains commands that set up the necessary options to recompute the current plot *exactly* as a printable plot. Note that this option requires the recomputation of the pseudospectra data, as well as access to the original matrix. |
| Save for Quick Print Creation | This option is similar to above, except that it saves the necessary data for re-creating the current pseudospectra in a .mat file. A printable plot can then be created almost instantly by calling EigTool with a single argument, the name of the file the data was saved in. |
| Projection Level | This parameter controls the amount of projection to use when computing pseudospectra; see the Projection Tutorial for information on choosing good values. |
| Colour | Toggle colour or black lines for the pseudospectra, useful if preparing plots for publication. |
| Thick Lines | Toggle thick or thin lines; thick lines are better for presentations (especially online, where a data projector is being used), whilst thin ones are better for publication plots. |
| Arbitrary Levels | Allows the use of epsilon levels which are not equally spaced, and hence cannot be entered using EigTool's standard Contour Levels pane. |
| Accuracy of FoV Comp. | By default the field of values (numerical range) is computed by connecting 40 points on its exterior using straight lines. This option allows the default value to be changed if finer resolution is required (the number must be even). |
| Display Eigenvalues | For some plots it may be desirable to remove the eigenvalues (for example if it is known that the pseudospectra are correct for a particular discretisation, but the eigenvalues are not). |
| Display Pseudospectra | Similarly, there may be cases where only the eigenvalues are of interest, and the pseudospectra need to be turned off. |
| Display Dimension | Displays the dimension of the matrix in the bottom left hand corner of the axes. This is useful for determining the size of the projected matrix if projection is being used. |
| Display Gridpoints | Displays the grid used for the pseudospectra computation; EigTool computes the norm of the resolvent inv(z\*I-A) at points z in a grid over the current portion of the complex plane. |
| Display Imaginary Axis | This item toggles the display of a grey line on the imaginary axis, useful for determing the stability of certain operators. |
| Display Unit Circle | This item toggles the display of a grey line on the unit circle, useful for determing convergence properties in certain applications. |
| Display Colourbar | This item toggles the display of the colourbar showing the different epsilon levels plotted by EigTool. This is especially useful for creating printable plots without the colourbar. |

# Some specific examples

## Options Code for Printing

As an example of creating options code, we will show how we created
the options for the Orr-Sommerfeld demo from the Demos menu. The following commands can be used
to create the matrix and start EigTool:

A =
orrsommerfeld\_demo(100);  
 eigtool(A);

The initial plot created by EigTool is shown below:

For this discretisation, it is known that only the rightmost
eigenvalues approximate those of the operator, so we change the axes
using either the text boxes or the mouse to focus on the area we
want. Adding the imaginary axis allows
us to see whether this operator is stable (which side of the line does
the rightmost eigenvalue lie on?), and increasing the number of grid
points to 50 gives a smooth plot:

If the Options Code for Printing menu option is now selected, the
following text is output in the main MATLAB window:

% Run the following commands to set up the necessary  
% options to create a printable plot of the pseudospectra.  
% Create the plot using 'eigtool(A,opts)' where A is your matrix.  
% (If you are using eigs, simply pass these options along with  
% the eigs options to eigs itself.)  
opts.npts=50;  
opts.levels=-7.5:0.5:-2;  
opts.ax=[-1 0.2 -1.05 0];  
opts.proj\_lev=Inf;  
opts.colour=1;  
opts.thick\_lines=1;  
opts.scale\_equal=1;  
opts.grid=0;  
opts.dim=1;  
opts.no\_ews=0;  
opts.no\_psa=0;  
opts.fov=0;  
opts.direct=1;  
opts.print\_plot=1;

To use this, copy it and paste it into a script (or the MATLAB
window), and add the lines

A = orrsommerfeld\_demo(100);  
eigtool(A,opts);

Running this script will then create a printable plot of the
eigenvalues and pseudospectra exactly as above:

Note that if you want to be left with an EigTool session rather
than a printable plot, simply set opts.print\_plot=0. For full
details of the options available, please see Command
Line Options.

## Save for Quick Print Creation

For some problems, the time taken to compute the pseudospectra data
may be substantial, and so it is desirable to have a method of saving
the computed data needed to create the plot, rather than the options
used as above. If we use this menu option to save the data into a file
called, say, os\_test.mat, the printable plot below (which is
identical to that above) can be instantly created using the command:

eigtool('os\_test');

## Arbitrary Levels

The exponentially spaced epsilon levels used as default by EigTool
are generally good, but for some problems more user control may be
desired. The Aribtrary Levels option is there to provide this by
allowing the user to enter their own levels via a text box:

In this example, the Random Demo is
shown, but for this near-normal matrix equally spaced levels may be
more appropriate:

Notice that the levels shown in the colourbar are no longer equally
spaced as an indication that non-exponentially spaced levels are being used.

---

EigTool home page.
