# Supplementary material for: Predictive analytics of environmental adaptability in multi-omic network models
Source: Sci Rep. 2015 Oct 20;5:15147. doi: 10.1038/srep15147 (PMC4611489; doi:10.1038/srep15147)
Supplement: Supplementary Information [file srep15147-s5.zip › source code METRADE/5) pseudospectra/eigtoollib/html/eigtool/documentation/menus/file.html]

EigTool File Menu


# File Menu

If a menu item in the table below is a hyperlink, click to see
examples.

|  |  |  |
| --- | --- | --- |
| New Matrix... |  | Use this item to bring a new matrix into EigTool. You will be prompted to enter a MATLAB command to create the matrix, or the variable name of the matrix in the base workspace. |
| Save Current State |  | This option allows you to save the entire current session of EigTool as a MATLAB figure, which you can then reload using the MATLAB open command. |
| Save Current State As... |  | This allows you to save the entire current session of EigTool under another name. |
| Export | Eigenvalues | Save the eigenvalues of the current matrix to a variable in the main MATLAB workspace. You will be prompted for a variable name to use. |
|  | Original Matrix | Save the original matrix to a variable in the main MATLAB workspace. You will be prompted for a variable name to use. This is useful if, for example, you call EigTool from within a script and later want to access the matrix, or create the matrix on-the-fly using the New Matrix menu item. |
|  | Pseudospectra Data | Save the pseudospectra data (vectors x and y defining the grid, and the singular value data) to variables in the main MATLAB workspace. You will be prompted for the variable names to use. |
|  | Schur Factor | Save the triangular Schur factor of the current matrix to the main MATLAB workspace. You will be prompted for a variable names to use. |
| Printable Plot... |  | Create a duplicate figure of the eigenvalues and pseudospectra without any of EigTool's buttons or controls. You will be prompted for a figure number that should be left blank in most cases; a new figure will then be created. If you wish to superimpose the pseudospectra onto an existing plot, you can enter the figure number to use here (ensuring that hold on has been set first). |
| Quit |  | Exit EigTool. |

# Some specific examples

## New Matrix...

Any valid MATLAB command can be entered when bringing in a new matrix,
although sequences of commands are not allowed. The following example
will create a Jordan block of dimension 20:

## Printable Plot...

In the following example, we first describe how the Printable Plot
option works, and then show how it can be used to superimpose
eigenvalues and pseudospectra onto an existing plot. The matrix we use
is the Grcar matrix of dimension 100 (available through the Demos menu), and we begin with EigTool as
follows:

Selecting Printable Plot and
clicking on the OK button (without entering any text) leaves us with
the following figure:

If instead of just
removing EigTool's buttons we want to add the EigTool plot to another
figure, we simply enter the number of the figure (in this case 2) into
the text box when prompted by Printable Plot. Note that we must first
ensure that hold on has been set, or the figure contents will
be overwritten. The figure below contains the spectrum of the
corresponding infinite dimensional Grcar matrix, the entire grey
region:

Here is the output produced by EigTool when the pseudospectra are
superimposed on top of it.:

Notice how the size and axes for the figure are determined by the
EigTool plot, not those that were already being used for the figure.

---

EigTool home page.
