# Supplementary material for: Predictive analytics of environmental adaptability in multi-omic network models
Source: Sci Rep. 2015 Oct 20;5:15147. doi: 10.1038/srep15147 (PMC4611489; doi:10.1038/srep15147)
Supplement: Supplementary Information [file srep15147-s5.zip › source code METRADE/5) pseudospectra/eigtoollib/html/eigtool/documentation/menus/frank_demo.html]

EigTool Frank Demo


# EigTool Frank Demo

The Frank matrix is also in the MATLAB gallery, and can be
created by the command

F = gallery('frank',N)

This is, a classic example of a matrix whose
eigenvalues are ill-conditioned and hence difficult to
compute [1,2]. What it illustrates is that one cannot
expect to compute eigenvalues better than a set of
eps-pseudoeigenvalues for some eps on the order of
machine precision. This is seen at the left of the
spectrum, where some eigenvalues are complex, even
though all the eigenvalues can be proved to be real. The
pseudospectra are shown in [3].

[1]: G. H. Golub and J. H. Wilkinson, "Ill-conditioned
eigensystems and the computation of the Jordan
canonical form", SIAM Review 18, pp. 578-619, 1976.

[2]: J. H. Wilkinson, "The Algebraic Eigenvalue Problem",
Clarendon Press, Oxford, 1965.

[3]: L. N. Trefethen, "Psuedospectra of matrices", in
"Numerical Analysis 1991" (Dundee 1991), Longman Sci.
Tech., Harlow, 1992, 234-266.

---

EigTool EigTool Demos Menu.
