# Supplementary material for: Predictive analytics of environmental adaptability in multi-omic network models
Source: Sci Rep. 2015 Oct 20;5:15147. doi: 10.1038/srep15147 (PMC4611489; doi:10.1038/srep15147)
Supplement: Supplementary Information [file srep15147-s5.zip › source code METRADE/5) pseudospectra/eigtoollib/html/eigtool/documentation/menus/gallery5_demo.html]

EigTool Gallery 5 Demo


# EigTool Gallery 5 Demo

This is matrix 5 from the MATLAB gallery: "an interesting
eigenvalue problem. Try to find its EXACT eigenvalues
and eigenvectors".

---

EigTool EigTool Demos Menu.
