# Supplementary material for: Predictive analytics of environmental adaptability in multi-omic network models
Source: Sci Rep. 2015 Oct 20;5:15147. doi: 10.1038/srep15147 (PMC4611489; doi:10.1038/srep15147)
Supplement: Supplementary Information [file srep15147-s5.zip › source code METRADE/5) pseudospectra/eigtoollib/html/eigtool/documentation/menus/godunov_demo.html]

EigTool Godunov Demo


# EigTool Godunov Demo

The "Godunov matrix" is a 7 x 7 matrix that has been used
by S. K. Godunov to illustrate the difficulty of computing
certain eigenvalues in floating point arithmetic [1]. The
entries of A are integers and the exact eigenvalues
are -4, -2, -1, 0, 1, 2, 4. This can be seen from the
fact that L\*A/L is upper triangular, where L is the
lower-triangular matrix defined by

L = eye(7); L(3,1) = 1; L(6,1) = 1;L(5,3) = 1; L(7,[2 3 5]) = 1;

[1]: S. K. Godunov, "Modern Aspects of Linear Algebra",
Translations of Mathematical Monographs v. 175,
Amer. Math. Soc., Providence, RI, 1998.

---

EigTool EigTool Demos Menu.
