# Supplementary material for: Predictive analytics of environmental adaptability in multi-omic network models
Source: Sci Rep. 2015 Oct 20;5:15147. doi: 10.1038/srep15147 (PMC4611489; doi:10.1038/srep15147)
Supplement: Supplementary Information [file srep15147-s5.zip › source code METRADE/5) pseudospectra/eigtoollib/html/eigtool/documentation/menus/grcar_demo.html]

EigTool Grcar Demo


# EigTool Grcar Demo

The Grcar matrix is also in the MATLAB gallery, and can be
created by the command

G = gallery('grcar',N)

This is a popular example in the field of matrix iterations
of a matrix whose spectrum is in the right half-plane but
whose numerical range is not. It's also a popular example
in the study of nonsymmetric Toeplitz matrices. The matrix
was first described in [1] and its pseudospectra were first
plotted in [2].

[1]: J. F. Grcar, "Operator coefficient methods for linear
equations", tech. report SAND89-8691, Sandia National
Labs, 1989

[2]: L. N. Trefethen, "Psuedospectra of matrices", in
"Numerical Analysis 1991" (Dundee 1991), Longman Sci.
Tech., Harlow, 1992, 234-266.

---

EigTool EigTool Demos Menu.
