# Supplementary material for: Predictive analytics of environmental adaptability in multi-omic network models
Source: Sci Rep. 2015 Oct 20;5:15147. doi: 10.1038/srep15147 (PMC4611489; doi:10.1038/srep15147)
Supplement: Supplementary Information [file srep15147-s5.zip › source code METRADE/5) pseudospectra/eigtoollib/html/eigtool/documentation/menus/gs_demo.html]

EigTool Gauss-Seidel Demo


# EigTool Gauss-Seidel Demo

Gauss-Seidel is one of the "classic matrix iterations" analyzed first by
Frankel in 1950 and incorporated into a general theory by Young in the
early 1950s [1,2,3]. This demonstration applies it to three standard
model problems based on discretizing a simple 1D differential equation
by a tridiagonal matrix A = L+D+U. The Gauss-Seidel matrix G is
obtained when the lower half of this matrix, L+D, is split from the
upper half.

In the "classic" case A is the symmetric (-1,2,-1)/N^2 matrix
associated with the 1D Laplace equation. The matrix G is very strongly
non-normal. However, the non-normality affects the smaller
eigenvalues, not the largest one, which was shown by Frankel and Young
to be of order 1-N^2. This is why the Frankel-Young theory, even
though it looks only at eigenvalues, matches numerical experiments.

The "downwind" and "upwind" cases come from discretizing an
equation that has convection as well as diffusion. Now A is
nonsymmetric and we have a choice of whether the Gauss-Seidel iteration
sweeps with or against the "wind", i.e., whether the larger or the
smaller half of A is split from the rest. The two choices lead to
matrices G with identical eigenvalues but very different convergence
behaviors as measured by norms of powers of G. For the
downwind sweep, nonnormality causes little trouble and convergence
matches an eigenvalue prediction from the start.

For the upwind
sweep, nonnormality is dominant and there is a period of stagnation
before the iteration begins to converge.

The connection of these upwind-downwind effects to pseudospectra
was first pointed out in [4].

[1]: S. Frankel,"Convergence rates of iterative treatments
of partial differential equations", Math. Comp. 4 (1950), 65-75.

[2]: G. H. Golub and C. F. Van Loan, "Matrix Computations", 3rd ed.,
Johns Hopkins U. Press, Baltimore, 1996.

[3]: R. J. LeVeque and L. N. Trefethen, "Fourier analysis
of the SOR iteration," IMA J. Numer. Anal. 8 (1988), 273-279.

[4]: L. N. Trefethen, "Pseudospectra of matrices", in D. F. Griffiths
and G. A. Watson, eds., Numerical Analysis 1991, Longman Scientific
and Technical, Harlow, Essex, UK, 234-266, 1992.

---

EigTool EigTool Demos Menu.
