# Supplementary material for: Predictive analytics of environmental adaptability in multi-omic network models
Source: Sci Rep. 2015 Oct 20;5:15147. doi: 10.1038/srep15147 (PMC4611489; doi:10.1038/srep15147)
Supplement: Supplementary Information [file srep15147-s5.zip › source code METRADE/5) pseudospectra/eigtoollib/html/eigtool/documentation/menus/hatano_demo.html]

EigTool Hatano-Nelson Demo


# EigTool Hatano-Nelson Demo

Since the Nobel prize-winning work of P. Anderson in 1958, random
tridiagonal matrices have been famous as models of disordered
condensed matter quantum systems; their eigenvectors are
exponentially localized, and this is connected with lack of
transparency to light and conductivity to electrons of certain
1D physical systems [1]. The Hatano-Nelson model was proposed
in 1996 by two physicists as a contribution to the "nonhermitian
quantum mechanics" of certain systems in physics and biology [2].
This example matrix is tridiagonal and exponentially nonnormal;
pseudospectra such problems are analyzed in [3]. Hatano and
Nelson actually proposed the periodic analogue with nonzero
corner elements, which is much closer to normal.

[1]: P. Anderson, "Absence of diffusion in certain random lattices",
Phys. Rev., 109 (1958), 1492-1505.

[2]: N. Hatano and D. R. Nelson, "Localization transitions in non-
Hermitian quantum mechanics", Phys. Rev. Lett. 77 (1996), 570-573.

[3]: L. N. Trefethen, M. Contedini, and M. Embree, "Spectra,
pseudospectra, and localization for random bidiagonal matrices"
Comm. Pure Appl. Math. 54 (2001), 595-623.

---

EigTool EigTool Demos Menu.
