# Supplementary material for: Predictive analytics of environmental adaptability in multi-omic network models
Source: Sci Rep. 2015 Oct 20;5:15147. doi: 10.1038/srep15147 (PMC4611489; doi:10.1038/srep15147)
Supplement: Supplementary Information [file srep15147-s5.zip › source code METRADE/5) pseudospectra/eigtoollib/html/eigtool/documentation/menus/help.html]

EigTool Help Menu


# Help Menu

|  |  |
| --- | --- |
| EigTool Documentation | Access this documentation. |
| Pseudospectra Tutorial | Display information on how to run the Pseudospectra Tutorial, a tutorial to explain the general use and interpretation of pseudospectra. |
| EigTool Website | This option launches your default web browser and links to the EigTool website, where the latest version of the software can be downloaded from.\| Pseudospectra Gateway \| This option lanches your default web browser and links to the , a site which contains a wealth of information about pseudospectra. \| |

---

EigTool home page.
