# Supplementary material for: Predictive analytics of environmental adaptability in multi-omic network models
Source: Sci Rep. 2015 Oct 20;5:15147. doi: 10.1038/srep15147 (PMC4611489; doi:10.1038/srep15147)
Supplement: Supplementary Information [file srep15147-s5.zip › source code METRADE/5) pseudospectra/eigtoollib/html/eigtool/documentation/menus/index.html]

EigTool Menus


# EigTool Menus

There are seven menus in EigTool:

- File- Extras- ARPACK/eigs- Transients- Demos- Help

as well as the standard MATLAB Windows menu.

---

EigTool home page.
