# Supplementary material for: Predictive analytics of environmental adaptability in multi-omic network models
Source: Sci Rep. 2015 Oct 20;5:15147. doi: 10.1038/srep15147 (PMC4611489; doi:10.1038/srep15147)
Supplement: Supplementary Information [file srep15147-s5.zip › source code METRADE/5) pseudospectra/eigtoollib/html/eigtool/documentation/menus/kahan_demo.html]

EigTool Kahan Demo


# EigTool Kahan Demo

The Kahan matrix is also in the MATLAB gallery, and can be
created by the command

G = gallery('kahan',N)

Kahan's matrix [2] was devised to illustrate that QR
factorisation with column pivoting is not a fail-safe
method for determining the rank of the matrix. Rank
determination is related to the "distance to singularity"
of a matrix [1], or in other words, how large a perturbation
is needed to make the matrix singular. The pseudospectra are
shown in [3].

[1]: N. J. Higham, "Matrix nearness problems and applications",
NA Rep. 161, Dept. of Maths., U. of Manchester, June 1988.

[2]: W. Kahan, "Numerical linear algebra", Canad. Math. Bull. 9,
pp. 757-801, 1966.

[3]: L. N. Trefethen, "Psuedospectra of matrices", in
"Numerical Analysis 1991" (Dundee 1991), Longman Sci.
Tech., Harlow, 1992, 234-266.

---

EigTool EigTool Demos Menu.
