# Supplementary material for: Predictive analytics of environmental adaptability in multi-omic network models
Source: Sci Rep. 2015 Oct 20;5:15147. doi: 10.1038/srep15147 (PMC4611489; doi:10.1038/srep15147)
Supplement: Supplementary Information [file srep15147-s5.zip › source code METRADE/5) pseudospectra/eigtoollib/html/eigtool/documentation/menus/landau_demo.html]

EigTool Landau Demo


# EigTool Landau Demo

T represents an integral equation from laser theory investigated
by Landau [1] in one of the earliest applications of
pseudospectra.

[1]: H. J. Landau, "The notion of approximate eigenvalues
applied to an integral equation of laser theory",
Quart. Appl. Math. 35 (1977), pp. 165-172.

---

EigTool EigTool Demos Menu.
