# Supplementary material for: Predictive analytics of environmental adaptability in multi-omic network models
Source: Sci Rep. 2015 Oct 20;5:15147. doi: 10.1038/srep15147 (PMC4611489; doi:10.1038/srep15147)
Supplement: Supplementary Information [file srep15147-s5.zip › source code METRADE/5) pseudospectra/eigtoollib/html/eigtool/documentation/menus/markov_demo.html]

EigTool Markov Demo


# EigTool Markov Demo

This is a Markov chain transition matrix
for a random walk on an N by N triangular lattice. It is an
example of a matrix for which certain parts of the spectrum
(in this case the interior) are highly non-normal, but other
eigenvalues are well-conditioned and relevant to the application.
(Here, the left eigenvectors associated with eigenvalues of unit
modulus determine the steady-state distribution of the Markov chain.
For more information, see [1].

[1]: Y. Saad, "Numerical methods for large eigenvalue
problems", Manchester University Press, Manchester,
p48ff, 1992.

---

EigTool EigTool Demos Menu.
