# Supplementary material for: Predictive analytics of environmental adaptability in multi-omic network models
Source: Sci Rep. 2015 Oct 20;5:15147. doi: 10.1038/srep15147 (PMC4611489; doi:10.1038/srep15147)
Supplement: Supplementary Information [file srep15147-s5.zip › source code METRADE/5) pseudospectra/eigtoollib/html/eigtool/documentation/menus/numbers.html]

EigTool Numbers Menu


# Numbers Menu

The Numbers menu is created dynamically when EigTool is started, using
files contained in the num\_comp directory, under the
eigtool directory. For some items, a point may be marked on
the pseudospectra plot, indicating where this numerical value is
attained (see Display Points). In these cases,
the point can be removed from the plot by selecting the corresponding
menu item a second time (a check mark by the item indicates whether
the point is displayed or not).

|  |  |
| --- | --- |
| Eigenvector Matrix Cond. No. | Compute the (2-norm) condition number of the matrix of eigenvectors. |
| Departure from Normality | This option will compute Henrici's *departure from normality* for the current matrix. This is defined to be the Frobenius norm of N where N = T-diag(l_i_), T is the Schur factor of the matrix and diag(l_i_) is the diagonal of T. |
| Numerical Abscissa | Compute the *numerical abscissa*, the rightmost point in the field of values. |
| Pseudospectral Abscissa | Compute (for a particular value of eps) the *pseudospectral abscissa*, the real part of the rightmost point in the eps-pseudospectrum. |
| Pseudospectral Radius | Compute (for a particular value of eps) the *pseudospectral radius*, the absolute value of the point with greatest modulus in the eps-pseudospectrum. |
| Spectral Abscissa | Compute the *spectral abscissa*, the real part of the rightmost eigenvalue of the matrix. |
| Spectral Radius | Compute the *spectral radius*, the absolute value of the eigenvalue with greatest modulus. |
| Display Points | When this option is toggled on, the points in the complex plane at which the above numbers are attained, if appropriate, are marked on the pseudospectra plot. When this item is toggled off, any existing points are removed, and no more will be plotted until it is toggled again. |

---

EigTool home page.
