# Supplementary material for: Predictive analytics of environmental adaptability in multi-omic network models
Source: Sci Rep. 2015 Oct 20;5:15147. doi: 10.1038/srep15147 (PMC4611489; doi:10.1038/srep15147)
Supplement: Supplementary Information [file srep15147-s5.zip › source code METRADE/5) pseudospectra/eigtoollib/html/eigtool/documentation/menus/olmstead_demo.html]

EigTool Olmstead Demo


# EigTool Olmstead Demo

This demo is the Matrix Market Olmstead
matrix (N can be 500 or 1000).
For more information, please see

http://math.nist.gov/MatrixMarket/data/NEP/olmstead/olmstead.html

---

EigTool EigTool Demos Menu.
