# Supplementary material for: Predictive analytics of environmental adaptability in multi-omic network models
Source: Sci Rep. 2015 Oct 20;5:15147. doi: 10.1038/srep15147 (PMC4611489; doi:10.1038/srep15147)
Supplement: Supplementary Information [file srep15147-s5.zip › source code METRADE/5) pseudospectra/eigtoollib/html/eigtool/documentation/menus/orrsomm_demo.html]

EigTool Orr-Sommerfeld Demo


# EigTool Orr-Sommerfeld Demo

This is an Orr-Sommerfeld operator generated from a spectral
method [2]. This discretization results in a matrix of
dimension N=99. There are numerous spurious eigenvalues
beyond the plotted axis, so the matrix is projected
onto an appropriate invariant subspace to accelerate
the computation. Pseudospectra of Orr-Sommerfeld
operators were first investigated in [1].

[1]: S. C. Reddy, P. J. Schmidt and D. S. Henningson,
"Pseudospectra of the Orr-Sommerfeld operator",
SIAM J. Appl. Math. 53(1), pp. 15-47, 1993.

[2]: L. N. Trefethen, "Spectral methods in MATLAB",
SIAM, 2000.

---

EigTool EigTool Demos Menu.
