# Supplementary material for: Predictive analytics of environmental adaptability in multi-omic network models
Source: Sci Rep. 2015 Oct 20;5:15147. doi: 10.1038/srep15147 (PMC4611489; doi:10.1038/srep15147)
Supplement: Supplementary Information [file srep15147-s5.zip › source code METRADE/5) pseudospectra/eigtoollib/html/eigtool/documentation/menus/pde900_demo.html]

EigTool Elliptic PDE Demo


# EigTool Elliptic PDE Demo

This demo is the Matrix Market PDE
matrix (N can be 900 or 2961).
For more information, please see

http://math.nist.gov/MatrixMarket/data/NEP/matpde/matpde.html

---

EigTool EigTool Demos Menu.
