# Supplementary material for: Predictive analytics of environmental adaptability in multi-omic network models
Source: Sci Rep. 2015 Oct 20;5:15147. doi: 10.1038/srep15147 (PMC4611489; doi:10.1038/srep15147)
Supplement: Supplementary Information [file srep15147-s5.zip › source code METRADE/5) pseudospectra/eigtoollib/html/eigtool/documentation/menus/randtri_demo.html]

EigTool Random Triangular Demo


# EigTool Random Triangular Demo

This is an upper triangular random matrix whose entries
are drawn from the normal distribution with mean 0 and
variance 1/N.

Unlike a matrix whose entries are all random, taking
non-zero entries only in the upper (or lower) triangle
produces a matrix with a high degree of non-normality [1,2].

[1]: J. M. Varah, "On the separation of two matrices",
SIAM J. Numer. Anal. 16, pp. 216-222, 1979.

[2]: L. N. Trefethen, "Psuedospectra of matrices", in
"Numerical Analysis 1991" (Dundee 1991), Longman Sci.
Tech., Harlow, 1992, 234-266.

---

EigTool EigTool Demos Menu.
