# Supplementary material for: Predictive analytics of environmental adaptability in multi-omic network models
Source: Sci Rep. 2015 Oct 20;5:15147. doi: 10.1038/srep15147 (PMC4611489; doi:10.1038/srep15147)
Supplement: Supplementary Information [file srep15147-s5.zip › source code METRADE/5) pseudospectra/eigtoollib/html/eigtool/documentation/menus/rand_demo.html]

EigTool Random Demo


# EigTool Random Demo

This is a random matrix whose entries are drawn from the
normal distribution with mean 0 and variance 1/N.

One can see from these pseudospectra that dense
random matrices are only mildly non-normal (in contrast,
for example, to triangular ones). In the limit N -> Inf,
the norm and spectral abscissa converge to 2 and 1,
respectively [1,2] and the condition number is of size
O(N) [3]. Of that factor of N, O(sqrt(N)) can be attributed
to variation in the size of the eigenvalues and O(sqrt(N))
to non-normality [4]. Pseudospectra for this example
were first plotted in [5].

[1]: S. Geman, "A limit theorem for the norm of random matrices"
The Annals of Probability 8(2), 1980, 252-261.

[2]: S. Geman, "The spectral radius of large random matrices"
The Annals of Probability 14(4), 1986, 1318-1328.

[3]: A. Edelman, "Eigenvalues and Condition Numbers of Random
Matrices", SIAM J. Matrix Anal. 9(4), 1988, 543-560.

[4]: J. T. Chalker and B. Mehlig, "Eigenvector Statistics in Non-
Hermitian Random Matrix Ensembles", Phys. Rev. Lett. 81(16),
1998, 3367-3370.

[5]: L. N. Trefethen, "Psuedospectra of matrices", in
"Numerical Analysis 1991" (Dundee 1991), Longman Sci.
Tech., Harlow, 1992, 234-266.

---

EigTool EigTool Demos Menu.
