# Supplementary material for: Predictive analytics of environmental adaptability in multi-omic network models
Source: Sci Rep. 2015 Oct 20;5:15147. doi: 10.1038/srep15147 (PMC4611489; doi:10.1038/srep15147)
Supplement: Supplementary Information [file srep15147-s5.zip › source code METRADE/5) pseudospectra/eigtoollib/html/eigtool/documentation/menus/riffle_demo.html]

EigTool Riffle Shuffle Demo


# EigTool Riffle Shuffle Demo

This matrix describes the Markov chain involved in riffle shuffling
of a deck of cards according to a model proposed by Gilbert, Shannon
and Reeds, and made famous by Diaconis and coauthors [1]. For a deck of
n cards, the state space is the set of all permutations, of dimension
factorial(n). Fortunately, as shown by Bayer and Diaconis, an
equivalent chain can be constructed on a state space of dimension just
n, the number of "rising sequences" in the deck. Formulas for
the entries of this n x n matrix P are essentially contained in [1]
and have been worked out explicitly by Gessel and Jonsson and Trefethen
[2].

Riffle shuffle is a famous example of a linear process with a strong
transient effect. Powers of P govern the approach to randomness, but
there is virtually no randomization at first followed by sudden
randomization later (asymptotically at the shuffle 1.5 log\_2(n)). Following
standard conventions in Markov chains, this code produces a matrix P
designed to act on row vectors on the left. To see the transient effect
with the limiting case P^inf subtracted off, look at the matrix A = P-Pinf
and its powers. The probabilistically correct norm in which to analyze
powers and pseudospectra is the 1-norm for the matrices as operating on
rows, which is the inf-norm according to standard Matlab conventions.
Thus pseudospectra, in particular, should in principle be examined in
the Matlab inf-norm, not the 2-norm utilized by EIGTOOL. Jonsson and
Trefethen found that these norms make little difference to the pseudospectra
(though the difference would be huge if one were working with the
underlying matrices of dimension n! rather than n).

[1]: D. Bayer and P. Diaconis, "Trailing the dovetail shuffle to its lair",
Annals Appl. Probab. 2, 1992, 294-313.

[2]: G. F. Jonsson and L. N. Trefethen, "A numerical analysis looks at the
`cut-off phenomenon' in card shuffling and other Markov chains", in
"Numerical Analysis 1997" (Dundee 1997), Addison Wesley Longman,
Harlow, 1998, 150-178.

---

EigTool EigTool Demos Menu.
