# Supplementary material for: Predictive analytics of environmental adaptability in multi-omic network models
Source: Sci Rep. 2015 Oct 20;5:15147. doi: 10.1038/srep15147 (PMC4611489; doi:10.1038/srep15147)
Supplement: Supplementary Information [file srep15147-s5.zip › source code METRADE/5) pseudospectra/eigtoollib/html/eigtool/documentation/menus/skewlap_demo.html]

EigTool Skew Laplacian Demo


# EigTool Skew Laplacian Demo

If the numbers 1.5 and 0.5 in the brief program below were both equal to 1,
it would generate the standard 7-point large sparse discretization of
the Laplace operator in three dimensions. With the numbers 1.5 and 0.5
as written, we get a highly nonnormal matrix with the same sparsity
pattern. A matrix like this might arise in the discretization of a
convection-diffusion problem.

I = speye(N-1);
  
D = N^2\*toeplitz([-2 1.5 zeros(1,N-3)],[-2 .5 zeros(1,N-3)]);
  
A = kron(I,kron(I,D)) + kron(I,kron(D,I)) + kron(D,kron(I,I));

---

EigTool EigTool Demos Menu.
