# Supplementary material for: Predictive analytics of environmental adaptability in multi-omic network models
Source: Sci Rep. 2015 Oct 20;5:15147. doi: 10.1038/srep15147 (PMC4611489; doi:10.1038/srep15147)
Supplement: Supplementary Information [file srep15147-s5.zip › source code METRADE/5) pseudospectra/eigtoollib/html/eigtool/documentation/menus/sprand_demo.html]

EigTool Sparse Random Demo


# EigTool Sparse Random Demo

This is a bidiagonal matrix with exponentially decaying entries on
the diagonal and 0.5 on the superdiagonal; a small amount of random
noise is then added throughout the matrix. This gives it a dense
spectrum in a ball around the origin with a few well separated
eigenvalues. It was used in [1] as an example of a large matrix for
which the pseudospectra of the projected matrix created during the
implicitly restarted Arnoldi iteration (ARPACK and eigs) are a good
approximation to the pseudospectra of the full matrix. In [1], the
dimension N = 200,000 and 30 eigenvalues were requested from a subspace
of dimension 50.

[1]: T. G. Wright and L. N. Trefethen, "Computation of pseudospectra
using ARPACK and eigs", SIAM J. Sci. Comp., 23(2), 2001, 591-605

---

EigTool EigTool Demos Menu.
