# Supplementary material for: Predictive analytics of environmental adaptability in multi-omic network models
Source: Sci Rep. 2015 Oct 20;5:15147. doi: 10.1038/srep15147 (PMC4611489; doi:10.1038/srep15147)
Supplement: Supplementary Information [file srep15147-s5.zip › source code METRADE/5) pseudospectra/eigtoollib/html/eigtool/documentation/menus/supg_demo.html]

EigTool SUPG Demo


# EigTool SUPG Demo

This is a matrix corresponding to the
SUPG discretisation of an advection-diffusion operator.
This demo is based on a function by Mark Embree, which
was essentially distilled from the IFISS software of
Elman and Silvester. See: http://www.ma.umist.ac.uk/djs/software.html
and [1].

For this example, whilst the eigenvalues computed by eigs
are inaccurate due to the sensitivity of the problem, the
approximate pseudospectra are very good approximations to
the true ones.

[1]: B. Fischer, A. Ramage, D. Silvester and A. Wathen,
"Towards Parameter-Free Streamline Upwinding for
Advection-Diffusion Problems", Comput. Methods Appl.
Mech. Eng., 179, 1999, 185-202.

---

EigTool EigTool Demos Menu.
