# Supplementary material for: Predictive analytics of environmental adaptability in multi-omic network models
Source: Sci Rep. 2015 Oct 20;5:15147. doi: 10.1038/srep15147 (PMC4611489; doi:10.1038/srep15147)
Supplement: Supplementary Information [file srep15147-s5.zip › source code METRADE/5) pseudospectra/eigtoollib/html/eigtool/documentation/menus/transients.html]

EigTool Transients Menu


# Transients Menu

If a menu item in the table below is a hyperlink, click to see
examples.

|  |  |
| --- | --- |
| Matrix Powers | Create a new figure and plot \|\|A^k\|\| against k. A lower bound computed using the spectral radius will be shown as a black dashed line. Press the Stop button in that figure to terminate the computation. The lower bound (spectral radius)^k is drawn as a black dashed line. |
| Matrix Exponentials | Create a new figure and plot \|\|exp(tA)\|\| against t. A lower bound computed using the spectral abscissa will be shown as a black dashed line. Press the Stop button in that figure to terminate the computation. The lower bound exp(t\*(spectral abscissa)) is drawn as a black dashed line. |
| Compute a Bound | Compute a lower bound based on the resolvent norm (pseudospectra) at a point you will be asked to select. This will be plotted as a green line on the Transient plot. |
| Best Estimate Lower Bound | Select the best lower bound based on all of the gridpoints used for the pseudospectra computation. |
| Information | Display a description of the lower bound computed using the above option. |

# Some specific examples

For these examples we use the Transient Demo, a matrix that is constructed to have
transient behaviour in both its powers and exponentials. The
eigenvalues and pseudospectra are shown below:

Note that the imaginary axis and unit circle are shown: these can be
turned on using the menu options.

## Matrix Powers

If we look at the norms of the powers of this matrix, we get the
following plot after a few seconds of computation:

Although the largest eigenvalue (in absolute value) predicts the
decay given by the black dashed line (it is inside the unit circle),
the norm of the powers grows over one hundredfold before
decaying as expected from the spectral radius [abscissa].

## Matrix Exponentials

If we look at the norms of the matrix exponential exp(t\*A) for various
values of t, we get the
following plot after a few seconds of computation. When prompted, we
chose a time step dt=2:

Although the largest real part of an eigenvalue predicts the decay
given by the black dashed line (it is in the left half-plane), the
norm grows over one hundredfold before decaying according to the
theory.

## Compute a Bound

Using pseudospectra, it is possible to compute lower bounds on the
size of this transient growth. After selecting this option, you will
be asked to click on a point in the complex plane to base the bound
upon; the bound is then computed as follows [1], where R is the resolvent
norm at the selected point z. The bound will be computed over the
values of k (or t) currently visable on the transient plot

**For matrix powers:**

max_{1<=k<=K}_||A^k|| >=
r^K/(1+(r^K-1)/((r-1)\*(rR-1)))  
             (only valid for |z| = r > 1).

**For matrix exponentials:**

sup_{0<=t<=T}_||exp(t\*A)|| >=exp(a\*T)/(1+(exp(a\*T)-1)/(aR))  
             (only valid for Re(z) = a > 0).

Clicking on a point near z=0.2 gives the following plot:

The bound appears as a green curve on the transient plot (with the
selected point highlighted on the pseudospectra plot). For any
particular point on this curve at step K (or time T), the bound says
that the transient growth must have been at least this large for some
point k < K (or t < T).

[1]: L. N. Trefethen, 2002, unpublished note

## Best Estimate Lower Bound

As well as allowing the user to select a point to base their bound
upon (see Compute a Bound), a best
estimate of the lower bound over *all* of the points in the grid
used for the current pseudospectra can be computed:

In general, this bound is usually within an order of magnitude of
the true growth.

---

EigTool home page.
