# Supplementary material for: Predictive analytics of environmental adaptability in multi-omic network models
Source: Sci Rep. 2015 Oct 20;5:15147. doi: 10.1038/srep15147 (PMC4611489; doi:10.1038/srep15147)
Supplement: Supplementary Information [file srep15147-s5.zip › source code METRADE/5) pseudospectra/eigtoollib/html/eigtool/documentation/menus/transient_demo.html]

EigTool Transient Demo


# EigTool Transient Demo

The matrix in this demo is designed to have transient
behaviour in both ||A^k|| and ||e^{tA}||. To see this,
run the demo, then go to the `Transients' menu to view
the behaviour. Lower bounds on the transient behaviour
can be plotted by selecting items from the same menu.

---

EigTool EigTool Demos Menu.
