# Supplementary material for: Predictive analytics of environmental adaptability in multi-omic network models
Source: Sci Rep. 2015 Oct 20;5:15147. doi: 10.1038/srep15147 (PMC4611489; doi:10.1038/srep15147)
Supplement: Supplementary Information [file srep15147-s5.zip › source code METRADE/5) pseudospectra/eigtoollib/html/eigtool/documentation/menus/twisted_demo.html]

EigTool Twisted Toeplitz Demo


# EigTool Twisted Toeplitz Demo

The "cross" matrix is an example of a banded "twisted circulant matrix".
It is tridiagonal (with periodic wraparound) but instead of having
constant diagonals, as in a true circulant matrix, it has a smoothly
varying main diagonal. The result is an exponentially strong degree
of nonnormality, with pseudomodes in the form of wave packets.
See [1].

[1]: J. Chapman and L. N. Trefethen, "Wave packet pseudomodes of twisted
Toeplitz matrices", to appear.

---

EigTool EigTool Demos Menu.
