# Supplementary material for: Predictive analytics of environmental adaptability in multi-omic network models
Source: Sci Rep. 2015 Oct 20;5:15147. doi: 10.1038/srep15147 (PMC4611489; doi:10.1038/srep15147)
Supplement: Supplementary Information [file srep15147-s5.zip › source code METRADE/5) pseudospectra/eigtoollib/html/eigtool/documentation/options/index.html]

EigTool Command Line Options


# EigTool Command Line Options

The contents of this page is summarised in the eigtool help
text, which can be seen by typing help eigtool at the MATLAB
command prompt.

There are several different combinations of command line arguments
that can be used with EigTool

## Basic EigTool Operation

The most basic way to run EigTool is simply to type eigtool
at the MATLAB command prompt. EigTool will then be started, and will
wait for you to run a Demo from the Demos Menu, or input a New Matrix.

If you already have a matrix (say A) you wish to use
EigTool for, simply type eigtool(A) and the eigenvalues and
pseudospectra will be computed automatically; depending on the Matrix Type, you may be asked some
questions before the computation begins.

## Using OPTIONS to Control the Plot

EigTool has many options available to control the final plot and the
computation method used. These are particularly useful when writing
scripts to call EigTool automatically as part of another
calculation. To run EigTool with options, type
eigtool(A,opts), where the options allowed are as follows:

|  |  |  |
| --- | --- | --- |
| **Option** | **Description** | **Value and {Default}** |
| opts.npts | number of gridpoints to use in the pseudospectra computation (if the axes are not square, this is the number used in longer direction; the number in the shorter direction will be in proportion to the aspect ratio of the axes); see Mesh | [scalar \| {automatic}] |
| opts.levels | log10(eps) for desired eps-pseudospectra to plot; see Contour Levels | [vector \| {automatic}] |
| opts.ax | axes on which to compute the pseudospectra/display the eigenvalues; see Figure Axes | [4-by-1 vector \| {automatic}] |
| opts.proj\_lev | pojection level to use (0..Inf); see the Projection Tutorial and Projection Level | [scalar \| {Inf}] |
| opts.colour or  opts.color | draw the pseudospectra using colored lines. See Colour | [0 \| {1}] |
| opts.thick\_lines | draw the pseudospectra using thick lines; see Thick Lines | [0 \| {1}] |
| opts.scale\_equal | use `axis equal' on axes; see Figure Axes | [0 \| {1}] |
| opts.print\_plot | only create a plot with eigenvalues and pseudospectra (no user interface controls); see Printable Plot | [{0} \| 1] |
| opts.no\_graphics | compute singular value data only, i.e., no user interface or graphical output | [{0} \| 1] |
| opts.no\_waitbar | suppress display of waitbar during computations (useful if calling EigTool from within a script) | [{0} \| 1] |
| opts.isreal | is A (complex) unitarily similar to a real matrix? If so, pseudospectra will be symmetric about the real axes, saving as much as a factor of two (depending on the axes) in the pseudospectra computation time. Useful if A was obtained from a Schur decomposition. | [{0} \| 1] |
| opts.ews | eigenvalues of rectangular or sparse matrices are not computed by default in EigTool (unless using ARPACK/eigs); if they are known in advance, they can be added to the plot using this option | [vector \| {[]}] |
| opts.dim | display the matrix dimension on the plot; see Display Dimension | [0 \| {1}] |
| opts.grid | display the grid as dots on the plot; see Display Grid | [{0} \| 1] |
| opts.no\_ews | suppress display of the eigenvalues (plot pseudospectra only); see Display Eigenvalues | [{0} \| 1] |
| opts.no\_psa | suppress display of the pseudospectra (plot eigenvalues only); see Display Pseudospectra | [{0} \| 1] |
| opts.fov | compute and display the field of values; see Field of Vals. | [{0} \| 1] |
| opts.unitary\_mtx | a unitary transformation to apply to eigenmodes and pseudoeigenmodes, useful if the input matrix was gererated from a unitary transformation of the original matrix. | [matrix \| {[]}] |
| opts.imag\_axis | highlight the imaginary axis in grey? See Display Imaginary Axis | [{0} \| 1] |
| opts.unit\_circle | highlight the unit circle in grey? See Display Unit Circle | [{0} \| 1] |
| opts.colourbar | Display the colourbar showing the epsilon levels plotted? See Display Colourbar | [{0} \| 1] |
| opts.direct | Use direct (rather than iterative) method; see Direct/Iterative | [0 \| {automatic}] |
| opts.k | No. of eigenvalues for eigs to search for. The default value is set by eigs, and is currently 6. | [1..n \| {automatic}] |
| opts.p | Maximum subspace size for eigs. The default value is determined by eigs, and is approximately twice the number of requested eigenvalues. | [1..n \| {default}] |
| opts.which | Which eigenvalues should eigs look for?. The default value is set by eigs, and is currently 'LM'. | [string\| {default}] |
| opts.tol | Tolerance for eigs to use. The default value is set by eigs, and is currently machine epsilon. | [scalar \| {default}] |
| opts.maxit | Maximum number of iterations for eigs. The default value is determined by eigs, and is max(300,2\*n/p). | [1..Inf \| {default}] |
| opts.v0 | Starting vector for eigs. The default value is determined by eigs, and is a random vector. | [vector \| {default}] |

## Advanced Calling Sequences

There are also a number of more advanced ways of calling EigTool:

eigtool(A,fig) will start EigTool in the figure number
fig.

eigtool(A,opts,fig) will start EigTool in the figure number
fig and use the options opts.

eigtool(data\_file) will create a Printable Plot from data saved
with Save for Quick Print Creation.

[x,y,sigs] = eigtool(A) will output the computed
pseudospectra data as vectors x and y defining the
grid, and sigs the singular value data.

eigtool(x,y,sigs) will create a Printable Plot using the
pseudospectra data x, y and sigs.

eigtool(x,y,sigs,fig) as above, but the plot is created in
figure number fig.

eigtool(x,y,sigs,opts) as above, but opts
contains options data to control the look of the plot. Note that not
all options are applicable here; for example, the axes are
determined by x and y, not opts.ax.

eigtool(x,y,sigs,opts,fig) as above, but the plot is created in
figure number fig using options opts.

---

EigTool home page.
