# Supplementary material for: Predictive analytics of environmental adaptability in multi-omic network models
Source: Sci Rep. 2015 Oct 20;5:15147. doi: 10.1038/srep15147 (PMC4611489; doi:10.1038/srep15147)
Supplement: Supplementary Information [file srep15147-s5.zip › source code METRADE/5) pseudospectra/eigtoollib/html/eigtool/documentation/options/prefs.html]

EigTool Command Line Options


# EigTool Preferences

All of EigTool's default options can be modified using MATLAB
preferences. For example, the default behaviour of EigTool displays
the matrix dimension on the plot unless this is overridden using command line options; some users may prefer that
the dimension is not displayed by default.

A full list of the default values that can be changed (and their
current settings) can be seen by typing getpref('EigTool') at
the MATLAB command prompt:

                  `version: 2.02  
                SMALL_GUI: 0  
                 colormap: [64x3 double]  
           levels_default: 'auto'  
               ax_default: 'auto'  
         proj_lev_default: Inf  
           colour_default: 1  
      thick_lines_default: 1  
      scale_equal_default: 1  
       print_plot_default: 0  
      no_graphics_default: 0  
       no_waitbar_default: 0  
              dim_default: 0  
             grid_default: 0  
    assign_output_default: 0  
              fov_default: 0  
      unitary_mtx_default: 1  
           no_ews_default: 0  
           no_psa_default: 0  
        imag_axis_default: 0  
      unit_circle_default: 0  
                p_default: 'auto'  
              tol_default: 'auto'  
               v0_default: 'auto'  
            which_default: 'LM'  
            maxit_default: 'auto'  
           direct_default: 'auto'  
            print_ew_size: 16  
        colourbar_default: 1  
         disp_pts_default: 'on'` 


### Setting a preference

Each preference can be changed using the command
setpref('EigTool','prefname',value) where prefname
is the name of the preference being changed, and value is the
new value.
Most of the preferences are the default values for the command line
options: see Command Line Options for a
description and possible values. The other preferences are described below:

|  |  |  |  |
| --- | --- | --- | --- |
| **Preference** | **Default** | **Possible values** | **Description** |
| version | 2.04 |  | The current EigTool version. DO NOT CHANGE THIS VALUE. |
| SMALL\_GUI | 0 | 0 or 1 | This switch is used to control the size of EigTool: large (0) for desktop machines, smaller for some laptops (1). |
| colormap | EigTool colourmap | Any MATLAB colourmap | The colourmap to be used by EigTool. This could be changed to jet for example, to produce pseudospectra with different colours. |
| disp\_pts\_default | 'on' | 'on' or 'off' | Display points when using items in the numbers menu automatically? |

### Resetting all the preferences

To reset the preferences to their default values, type the command
set\_eigtool\_prefs('default') at the MATLAB command prompt.

---

EigTool home page.
