# Supplementary material for: Predictive analytics of environmental adaptability in multi-omic network models
Source: Sci Rep. 2015 Oct 20;5:15147. doi: 10.1038/srep15147 (PMC4611489; doi:10.1038/srep15147)
Supplement: Supplementary Information [file srep15147-s5.zip › source code METRADE/5) pseudospectra/eigtoollib/html/eigtool/documentation/panes/index.html]

EigTool Panes


# EigTool Panes

If a menu item in the table below is a hyperlink, click to see
examples.

|  |  |
| --- | --- |
| Figure Axes | This pane allows control of the current axes. By default, the scale on the real and imaginary axes is equal, but if this is not required it can be turned off by toggling the check box Scale Equal. The axis limits can be controlled by entering text in the text boxes; the axes will change when the cursor leaves the box or the Return key is pressed. |
| Direct/Iterative | This pane controls whether a direct method (either dense or sparse, depending on the matrix type) or iterative method (eigs) is used. If eigs is being used, there are options to control the number of eigenvalues to request and the portion of the spectrum to focus on; for more eigs options, see the ARPACK/eigs menu. |
| Contour Levels | This is where the contour levels plotted are controlled from. The three text boxes allow you to enter the smallest and largest level to draw, and the jump between levels. The button Smart Levels will use EigTool's default levels. |
| Mesh | Use this text box to control the mesh size for the pseudospectra computation. A finer mesh will give a smoother picture (at least when printed as a postscript file, even if not on the screen), but the computation will take longer. |

# Some specific examples

## Direct/Iterative

For this example, we first compute the eigenvalues and pseudospectra
of the Basor-Morrison Toeplitz matrix from the Demos menu:

Now, instead of using a (dense) direct method for this 50 by 50
matrix, we can switch to using ARPACK/eigs, and ask for 15 eigenvalues
of largest real part:

Notice that when EigTool finishes computing the eigenvalues with
eigs, the pseudospectra based on the rectangular Hessenberg
matrix created during the Arnoldi iteration are plotted. These are
subsets of the true pseudospectra, as can be easily seen here.

## Contour Levels

For this example, we first compute the eigenvalues and pseudospectra
of the Random Triangular matrix from the Demos menu, and then zoom in on the
left portion of the spectrum using the mouse. After recomputing the
pseudospectra on this new region of the complex plane, we get the
following figure:

The contour levels we see are the same as those determined by EigTool
when the first pseudospectra plot was produced, on the original
axes. If we only want to consider levels appropriate to the axes we
now have after zooming, we can press the Smart Levels button to allow
EigTool to choose the appropriate levels for us:

The levels displayed using Smart Levels are determined by looking at
the range of singular values computed on the current grid and the size
of the current axes.

---

EigTool home page.
