# Supplementary material for: Predictive analytics of environmental adaptability in multi-omic network models
Source: Sci Rep. 2015 Oct 20;5:15147. doi: 10.1038/srep15147 (PMC4611489; doi:10.1038/srep15147)
Supplement: Supplementary Information [file srep15147-s5.zip › source code METRADE/5) pseudospectra/eigtoollib/html/eigtool/documentation/proj_tutorial/index.html]

Projection Tutorial


---

|  |  |  |
| --- | --- | --- |
|  | Start, Page 2, Page 3, Page 4, Page 5. | Next >> |

---

## Projection Tutorial

This tutorial is designed to take you through an example where the
the time needed to create a high-resolution publication quality plot
of the pseudospectra of a matrix is significantly reduced by
projection onto a carefully chosen invariant subspace.

This tutorial assumes that you have already downloaded the latest version of EigTool, and have
followed all of the installation instructions on that page.

The tutorial is organised into five parts:

- Step 1: Set up the data- Step 2: Select the portion of the complex plane of interest- Step 3: Begin to introduce projection- Step 4: Reduce the projection parameter still further- Step 5: Compute the pseudospectra on a fine grid of size 200 by 200

You can download the code used to generate the matrices used in
this tutorial by clicking here. This
is taken from Trefethen's paper, Computation of Pseudospectra (Acta
Numerica, 1999), which describes projection in greater detail.

- #### Step 1: Set up the data.

  Generate the matrix B by downloading the code and running it with N=200:

  B = lnt\_comp\_psa\_mtxs(200);

  Compute the pseudospectra using the default settings:

  eigtool(B)

  You should end up with something like the following image, which
  takes about 30 seconds to generate on my Sun Ultra 5 workstation.

  Note the large magnitudes of the x and y axes. Thes axes are chosen
  automatically by EigTool, and are this big because of
  the outlying eigenvalue near -80,000.

---

|  |  |  |
| --- | --- | --- |
|  | Start, Page 2, Page 3, Page 4, Page 5. | Next >> |

---

---

EigTool home page.
