# Supplementary material for: Predictive analytics of environmental adaptability in multi-omic network models
Source: Sci Rep. 2015 Oct 20;5:15147. doi: 10.1038/srep15147 (PMC4611489; doi:10.1038/srep15147)
Supplement: Supplementary Information [file srep15147-s5.zip › source code METRADE/5) pseudospectra/eigtoollib/html/eigtool/documentation/proj_tutorial/proj_tut_p2.html]

Projection Tutorial (page 2)


---

|  |  |  |
| --- | --- | --- |
| << Prev | Start, Page 2, Page 3, Page 4, Page 5. | Next >> |

---

## Projection Tutorial (page 2)

- #### Step 2: Select the portion of the complex plane of interest.

  This plot shows the pseudospectra in a region of the complex plane
  which contains the *entire* spectrum of the matrix B. Our
  interest, however, is in the rightmost eigenvalues, the ones with
  largest real part. To focus on these, we change the axis limits (using
  the text boxes towards the bottom left of EigTool) so that the real
  axis ranges from -100 to 50 and the imaginary axis from 0 to 150.

  After changing these limits, click on `Go!' to recompute the
  pseudospectra on this new domain. You will get a message saying that
  there are no contours to plot in the range specified, and that `Smart'
  levels have been used instead; this is because the original levels are
  no longer appropriate. You should end up with something
  like the following image, which my Sun Ultra 5 takes about 10 seconds
  to compute:

---

|  |  |  |
| --- | --- | --- |
| << Prev | Start, Page 2, Page 3, Page 4, Page 5. | Next >> |

---

---

EigTool home page.
