# Supplementary material for: Predictive analytics of environmental adaptability in multi-omic network models
Source: Sci Rep. 2015 Oct 20;5:15147. doi: 10.1038/srep15147 (PMC4611489; doi:10.1038/srep15147)
Supplement: Supplementary Information [file srep15147-s5.zip › source code METRADE/5) pseudospectra/eigtoollib/html/eigtool/documentation/proj_tutorial/proj_tut_p3.html]

Projection Tutorial (page 3)


---

|  |  |  |
| --- | --- | --- |
| << Prev | Start, Page 2, Page 3, Page 4, Page 5. | Next >> |

---

## Projection Tutorial (page 3)

- #### Step 3: Begin to introduce projection.

  The previous image is perfectly adequate for getting a rough idea
  of the behaviour of the pseudospectra near the imaginary axis, but it
  would be better to have a higher quality plot for inclusion in a
  publication or talk. The problem is that although this plot took only
  a few seconds to generate, it is only computed on a coarse 24 by 24
  grid (with 576 grid points in total). To move to a grid of 200 by 200 points
  to give a high quality plot would entail a wait of nearly twelve minutes
  on my Sun Ultra 5. This may be feasible for this reasonably
  small matrix, but we can do better.

  The key is to project our matrix of dimension 200 onto a subspace
  spanned by the invariant subspace corresponding to the eigenvalues
  near the region of the complex plane we are interested in. The
  projected matrix will hopefully have dimension much less than 200, and
  so the computation will be faster.

  The following figures indicate how our projection algorithm
  operates. The green square represents the area of the complex plane
  visible in EigTool (the area our grid is defined over), while the red
  square represents the eigenvalues whose eigenvectors we project
  onto. In the left figure, the projection level is set to 2,
  while in the right one the projection level is set to 1. The red
  rectangle is defined as the region which is (1+2b) times as high and
  (1+2b) times as wide as the rectangle visible in EigTool, where b is
  the projection parameter.

  |  |  |
  | --- | --- |
  |  |  |

  When you click `Go!', the matrix is first projected onto the space
  spanned by eigenvectors whose eigenvalues are within the red
  square. This is a non-trivial operation, but the benefits will far
  outweigh this extra cost if we are computing on a fine grid. The
  pseudospectra of this smaller, projected matrix are then computed. As
  long as the effect of the eigenvalues we have left out of our
  projection is small, the plot of the pseudospectra will look
  essentially identical, and importantly, the computation will be
  faster.

  First, try reducing the projection level to 2 using the Projection Level option of the
  Extras menu, and recomputing the pseudospectra. The plot you get
  should look exactly the same as the one obtained before (with infinite
  projection level i.e. no projection). On the coarse grid used here, the
  computation time is worse (we have to take account of the time taken
  to project the matrix), but we will see real differences when we move
  to the fine grid. This takes about 25 seconds on my Sun Ultra 5
  workstation. Notice that the matrix dimension displayed by EigTool is
  now dim=200->126 not dim=200 (if you can't see this, turn on Display dimension in the Extras
  menu), indicating that the projected matrix has dimension 126.

  Since we saw no difference for a projection level of 2, now reduce
  it to 1 and recompute again. Once more, there is no change to the
  pseudospectra. This time the computation is quicker, since the matrix
  dimension has already been reduced during the first projection. Your
  EigTool should now look like this:

---

|  |  |  |
| --- | --- | --- |
| << Prev | Start, Page 2, Page 3, Page 4, Page 5. | Next >> |

---

---

EigTool home page.
