# Supplementary material for: Predictive analytics of environmental adaptability in multi-omic network models
Source: Sci Rep. 2015 Oct 20;5:15147. doi: 10.1038/srep15147 (PMC4611489; doi:10.1038/srep15147)
Supplement: Supplementary Information [file srep15147-s5.zip › source code METRADE/5) pseudospectra/eigtoollib/html/eigtool/documentation/proj_tutorial/proj_tut_p4.html]

Projection Tutorial (page 4)


---

|  |  |  |
| --- | --- | --- |
| << Prev | Start, Page 2, Page 3, Page 4, Page 5. | Next >> |

---

## Projection Tutorial (page 4)

- #### Step 4: Reduce the projection parameter still further.

  As there was once again no difference, try reducing the projection
  level again, this time to 0.5. If you look carefully at this plot, you
  can see that the pseudospectra are not the same as the previous plots
  at the left edge of the axes. If you reduce it still further, to 0.25,
  you should get the plot below, which again takes about 5 seconds to
  compute on my computer. The important thing to note here is that the
  pseudospectra of the projected matrix may be misleading when the
  invariant subspace that is projected upon makes a small angle with an
  eigenvector that is omitted from the projection; you must be careful
  not to project too far.

---

|  |  |  |
| --- | --- | --- |
| << Prev | Start, Page 2, Page 3, Page 4, Page 5. | Next >> |

---

---

EigTool home page.
