# Supplementary material for: Predictive analytics of environmental adaptability in multi-omic network models
Source: Sci Rep. 2015 Oct 20;5:15147. doi: 10.1038/srep15147 (PMC4611489; doi:10.1038/srep15147)
Supplement: Supplementary Information [file srep15147-s5.zip › source code METRADE/5) pseudospectra/eigtoollib/html/eigtool/documentation/proj_tutorial/proj_tut_p5.html]

Projection Tutorial (page 5)


---

|  |  |  |
| --- | --- | --- |
| << Prev | Start, Page 2, Page 3, Page 4, Page 5. |  |

---

## Projection Tutorial (page 5)

- #### Step 5: Compute the pseudospectra on a fine grid of size 200 by 200.

  First of all, set the projection parameter back to 1. Now set the number
  of gridpoints to 200, and hit the `Go!' button. The computation takes
  me about 3 minutes, less a quarter of the predicted time without
  projection. The final figure is shown below, and although the lines
  appear jagged on the screen, they will be smooth when printed out as a
  postscript file. Note that the matrix dimension is now just 92, less than
  half of the original size.

---

|  |  |  |
| --- | --- | --- |
| << Prev | Start, Page 2, Page 3, Page 4, Page 5. |  |

---

---

EigTool home page.
