# Supplementary material for: Predictive analytics of environmental adaptability in multi-omic network models
Source: Sci Rep. 2015 Oct 20;5:15147. doi: 10.1038/srep15147 (PMC4611489; doi:10.1038/srep15147)
Supplement: Supplementary Information [file srep15147-s5.zip › source code METRADE/5) pseudospectra/eigtoollib/html/eigtool/index.html]

Redirect to EigTool Homepage

You should automatically be forwarded to the EigTool homepage. If this does not
happen, please click http://www.comlab.ox.ac.uk/pseudospectra/eigtool/.
